# Attitudes Toward Asylum Policy in a Divided Europe: Diverging Contexts, Diverging Attitudes?

**DOI:** 10.3389/fsoc.2020.00035

**Published:** 2020-05-21

**Authors:** Arno Van Hootegem, Bart Meuleman, Koen Abts

**Affiliations:** ^1^Institute for Social and Political Opinion Research, Centre for Sociological Research, KU Leuven, Leuven, Belgium; ^2^Department of Sociology, Tilburg School of Social and Behavioral Sciences, Tilburg University, Tilburg, Netherlands

**Keywords:** attitudes toward asylum policy, economic context, policy context, migratory context, threat perceptions, human values, multilevel structural equation modeling

## Abstract

The large inflow of asylum-seekers in recent years has heralded a diversification in adopted asylum policies across European societies. Although a growing body of research has addressed these versatile approaches and their implications for the European integration project, insight into the social basis of these restrictive or open asylum policies remains underdeveloped. Hence, the current study provides detailed insight into public preferences for asylum policies and offers a new understanding of how these attitudes are affected by diverging socio-economic realities across Europe. In addition, this paper considers the role of individual factors that coincide with publicly adopted frames in contemporary asylum debates. In particular, to explain how contextual differences reflect on opinion climates, the impacts of the policy, economic, and migratory context are studied. On the individual-level, we focus on threat perception and human values, which represent humanitarian, economic, and cultural frames. To explore these relations, data on 20 countries from the European Social Survey Round 8 (2016) are analyzed through a multilevel structural equation modeling approach. Results indicate that, on the contextual-level, only unemployment rates have a significant impact and, rather surprisingly, lower unemployment rates provoke a more negative opinion climate. Yet, this relationship seems to be largely driven by some specific countries that are characterized by large unemployment rates and relatively positive opinion climates simultaneously. The migratory and policy context, on the other hand, do not influence attitudes toward asylum policy. This indicates that it is not necessarily the countries facing the largest inflow of asylum-seekers or issuing the most positive decisions on asylum applications that have the most restrictive opinion climates. As shown by the important roles of human values and threat perceptions, which represent widely adopted frames, public discourses seem much more important in explaining attitudes toward asylum policy across Europe.

## Introduction

The increased inflow of asylum seekers over the last years instigated fierce debates among European policy makers about the appropriate way to handle this new “crisis” (Hercowitz-Amir et al., 2017). As member states failed to agree on which rules to implement, a joint European reaction remained absent and the limits of the Common European Asylum System (CEAS) became apparent (Niemann and Zaun, 2018). Some countries, such as Germany, advocated for relocation schemes and a pragmatic response. Yet others, including the Visegrád countries, opposed the introduction of quota and the idea of burden-sharing (Castells, 2018; Glorius, 2018; Zaun, 2018). This lack of effective cooperation and the inability to develop harmonized asylum policies have intensified cleavages between states that pursue more restrictive policies, and nations that are more open and welcoming toward newcomers (Bakker et al., 2016; Castells, 2018; Zaun, 2018).

These opposing political reactions coincide with two broader conflicting perspectives on the desired design of asylum policies and the approach in handling the renewed inflow of asylum seekers (Triandafyllidou, 2018). On the one hand, the humanitarian perspective emphasizes the importance of open policies, a welcoming and solidary culture, and compassion with refugees and asylum seekers (De Cleen et al., 2017; Triandafyllidou, 2018). On the other hand, the exclusionary perspective advocates for the restricted admission of asylum seekers and understands the inflow of asylum seekers as an European crisis that is above all damaging to the well-being of the native population (De Cleen et al., 2017). This perspective has mainly been advocated by populist radical right parties across Europe.

While there is growing scholarly attention for these deepening political cleavages and their implications for the European integration project (Zaun, 2018), there is far less insight into whether this context has also instigated polarization between European populations in terms of attitudes toward humanitarian vs. exclusionary asylum policies. In the light of the current political divides, the question remains how arguments used on either side of the humanitarian-exclusionary spectrum are echoed in public opinion. Understanding popular attitudes toward asylum policy is crucial to grasp the dynamics of policy-making as well as the intergroup climates wherein asylum seekers have to be embedded. To remedy this knowledge gap, this study uncovers the preferences of European citizens for asylum policies that are aimed at either curbing the inflow or giving access to larger numbers of asylum seekers. While we recognize that asylum policies are multi-dimensional (Gest et al., 2014), we focus specifically on the opposition between openness and restrictiveness to understand how current policy debates in Europe resonate among the public at large.

Although previous research (Coenders et al., 2004; Ivarsflaten, 2005) has shown that a majority of the public does not oppose allowing refugees to stay in a given country, the current political context warrants deeper understanding of European citizens' attitudes toward asylum policies. The current situation differs profoundly in terms of the inflow rate of asylum seekers as well as in the cultural background of the majority of applicants compared to the previous peak in asylum applications that Europe faced in the aftermath of the Kosovo War (Heisbourg, 2015; OECD, 2015). However, our study does not only examine attitudes in this context but also tries to understand how contextual divisions between countries (for example in terms of economic situation and political context) reflect on public opinion. Particularly, we zoom in on the impact of structural factors that drive decisions on asylum policies and are regularly referred to in public debates.

In sum, our contribution aims to uncover how attitudes toward asylum policy take shape within the current social-economic context and how they are dependent of the various national contexts across Europe. In line with previous studies (Coenders et al., 2004; Ivarsflaten, 2005; Steele and Abdelaaty, 2018; Bolt and Wetsteijn, 2018; Heizmann and Ziller, 2019), we pay attention to individual as well as country-level determinants of attitudes toward asylum policies. We investigate the impact of cross-national differences in the policy, economic and migration context as well as individual-level mechanisms (basic human values and threat perceptions) that shape attitudes toward asylum policy. The individual-level factors are chosen so that each of them coincides with frames or arguments adopted in contemporary discussions on the asylum issue and, as a result, enable to comprehend the underlying mechanisms of the country-level effects. To study the impact of the individual and contextual determinants, we analyze data from the European Social Survey Round 8 (2016) by means of multilevel structural equation modeling (MSEM; Meuleman, 2019).

## Individual Attitudes Toward Asylum Policy: the Roles of Ethnic Threat and Human Values

Although attitudes toward various categories of migrants can empirically be strongly related and might partly stem from the same antecedents (Meuleman and Billiet, 2003; Meeusen et al., 2018), we study opinions regarding policies targeting asylum seekers separately. After all, the public can experience distinct forms of threat by various migrant groups (Meuleman et al., 2018) and differentiate in their attitudes toward them (Bansak et al., 2016; Czymara and Schmidt-Catran, 2017). These findings legitimize a separate analysis of attitudes toward asylum policies, especially in times of intensified debates on issues related to asylum seekers and refugees (e.g., relocation schemes, asylum centers, tenability of the Dublin regulation, …). Yet, to shed light on the (dis)similarity of policy preferences regarding asylum and migration in general, we also explicitly compare both attitudes.

Before turning to the discussion of the contextual factors that create dividing lines between countries—that is, the main focus of this paper—, we first discuss the impact of the individual-level mechanisms that underpin the formation of attitudes toward asylum policy. In particular, we focus on perceptions of economic and cultural threat as well as on the basic human values of universalism and conformity-tradition. Not only are each of these included factors important in shaping sentiments toward immigrants (McLaren, 2003; Davidov et al., 2014), they also relate closely to the main arguments or frames present in public discourses on asylum policies. On the one hand, certain frames stress a solidary or moral responsibility to take care of asylum seekers and refugees who are the main victims of the “crisis” (Triandafyllidou, 2018). This type of humanitarian discourse stresses the importance of aiding asylum seekers and installing a “*Willkommenskultur*” (De Cleen et al., 2017; Ritter and Rhomberg, 2017). On the other hand, threat frames are mobilized that represent the inflow of immigrants as disastrous for the well-being of the host population (Triandafyllidou, 2018). These arguments highlight the economic, cultural, and securitarian impact of admitting asylum seekers, as they focus on the costs for the economy, the potential erosion of dominant cultural values, and the endangerment of the safety of the native population, respectively (De Cleen et al., 2017; Ritter and Rhomberg, 2017). In absence of contextual data capturing the salience of the different discourses, this paper investigates whether individuals' endorsement of core messages of the various frames is related to policy preferences. Thus, instead of investigating how discourses influence attitudes toward asylum policy on the macro-level, we examine how opposition vs. support for generous policies crystallizes around attitudinal axes that relate to the frames—i.e., humanitarianism and (cultural, economic, or security) threat. When endorsement of particular frames is strongly predictive of attitudes toward asylum policy, we see this as an indication that the respective frame plays an important role in the public debate. Moreover, the proposed predictors are chosen so that they contribute to the understanding of how contextual differences reflect on public opinion. The investigated frames often refer to contextual factors (such as the economic situation and the number of asylum applications; cf. De Cleen et al., 2017; Ritter and Rhomberg, 2017) and the mechanisms behind these individual predictors are generalizable to the macro-level (cf. McLaren, 2003). Although we focus on the relationships between these variables and attitudes toward asylum policy and hence only formulate hypotheses about opinions on asylum, we investigate also whether these predictors operate similarly for attitudes toward immigration policy in general.

Group conflict theory (GCT) posits that negative attitudes of majority group members toward outgroups could be perceived as a reaction to ethnic competition over scarce resources (Coser, 1956; Blumer, 1958; Blalock, 1967; Bobo, 1983; Quillian, 1995). Previous studies found that perceived ethnic threat indeed leads to negative attitudes toward asylum seekers and preferences for restrictive asylum policies (Coenders et al., 2004; Bolt and Wetsteijn, 2018; Steele and Abdelaaty, 2018). Yet, these studies have not taken into account the distinction between competition over material resources (i.e., housing and welfare) and conflict over symbolic goods (i.e., norms, values, and identities; Meuleman et al., 2009). While the first source of competition sets of feelings of economic threat, the latter instigates cultural threat perceptions. Differentiating between both forms of threat is not only important because they refer to distinct ways of framing the inflow of asylum seekers (De Cleen et al., 2017; Greussing and Boomgaarden, 2017; Ritter and Rhomberg, 2017), but also because they differ in their antecedents (e.g., the impact of economic indicators) as well as consequences (e.g., voting behavior; Sniderman et al., 2004; Lucassen and Lubbers, 2011; Harell et al., 2012; Meuleman et al., 2017).

To begin with, individuals might feel economically threatened by the inflow of asylum seekers, as their presence is often portrayed as strengthening competition over scarce material resources (Ivarsflaten, 2005; Tartakovsky and Walsh, 2016). This fear coincides with an often-heard discourse that portrays asylum seekers as disguised economic migrants that seek to profit materially from the European welfare state (De Cleen et al., 2017; Ritter and Rhomberg, 2017). The economic frame builds on socio-tropic concerns about the financial impact of asylum seekers and sees them as a threat to the prosperity and welfare of the host country (Greussing and Boomgaarden, 2017; Ritter and Rhomberg, 2017). In addition, concerns that asylum seekers or immigrants with diverging normative views endanger national identity, such as the native language, religious practices, and dominant traditions, gives rise to cultural threat (Ivarsflaten, 2005; Schlueter et al., 2013). This cultural threat frame is frequently embedded in an rhetoric that identifies asylum seekers from Muslim countries as a source of “Islamization,” and defines Islam as being incompatible with “Western civilization” and its core liberal values, such as secularism and equality between men and women (Bracke, 2012; Czymara and Schmidt-Catran, 2017; De Cleen et al., 2017; Lucassen, 2018). Both forms of threat are embedded in negative discourses that stimulate the restriction of the admission of asylum seekers to limit economic competition and to preserve cultural traditions (Ivarsflaten, 2005). Hence, we expect that economic (*Hypothesis 1*) as well as cultural threat (*Hypothesis 2*) will lead to more support for restrictive asylum policies, although the strength of both effects might differ.

Yet according to GCT as well, economic and cultural threat perceptions are not distributed evenly across the population, but are socially stratified. In particular, especially low-status groups, such as lower educated and low-income individuals, are prone to feel economically and culturally threatened and to support restrictive policies, as they often compete more over the same material resources (Schneider, 2008) and have more limited cultural capital, which makes them less likely to value diversity and cultural differences (Manevska and Achterberg, 2013). Economic and cultural threat thus function as mediating factors in the relation between socio-economic status and attitudes toward asylum policy.

However, this GCT-based model exclusively focuses on ethnic competition and disregards the important role of basic human values. Previous research has evidenced how values, as trans-situational goals, are powerful factors shaping specific attitudes and evaluations of the social world (Sagiv and Schwartz, 1995; Davidov et al., 2008, 2014). The values are included not only because they are closely connected to publicly adopted frames on the cost and benefits of admitting asylum seekers (Tartakovsky and Walsh, 2016), but also because they correspond to norms indentifying appropriate behaviors and reactions to immigration-related dilemma's (cf. Ziller et al., 2019). In the case of intergroup attitudes, especially the values universalism and conformity-tradition are relevant. First, universalism emphasizes tolerance and the defense of the welfare of all human beings (Schwartz, 1994, p. 22). This value resonates within the humanitarian resettlement discourse that stresses the moral duty to protect needy asylum seekers (Schwartz, 2006; Davidov et al., 2014; Tartakovsky and Walsh, 2016; Ritter and Rhomberg, 2017). This discourse was prominent in the beginning of the “crisis” and conflicts with the primordial nationalistic frame that completely restricts the inclusion of newcomers (Roccas et al., 2010). Second, the value conformity-tradition refers to a commitment both to the compliance with social expectations and to the preservation of traditional culture (Schwartz, 1994, p. 22). This value coincides with a frame that portrays asylum seekers as disruptive for social order and threatening to social cohesion (Tartakovsky and Walsh, 2016). This logic is further reflected in political debates when the core liberal values of so-called Western civilization are protected to preserve national integrity (Lucassen, 2018). While universalism is expected to stimulate support for open asylum policies (*Hypothesis 3*), the internalization of conformity-tradition is anticipated to foster restrictive attitudes (*Hypothesis 4*).

**Figure 1:**
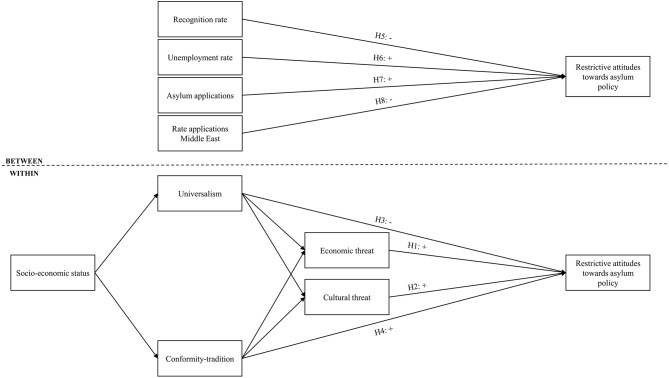
Conceptual model with hypotheses.

Simultaneously, universalism should lower perceived ethnic threat and conformity-tradition should increase cultural and economic fears (Sagiv and Schwartz, 1995; Davidov et al., 2014). We also expect that these values are socially stratified, as high-status individuals are more prone to internalize universalism and less likely to rely on conformity and tradition (Schwartz, 2007; Meuleman et al., 2013). Our theoretical model is thus a partial serial mediation, where the social structural characteristics influences human values that in turn impact threat perceptions, which eventually shape attitudes toward asylum policy. The conceptual model with the various hypotheses is visualized in Figure 1. We also estimate direct effects of the social structural variables on threat perceptions and attitudes toward asylum policy, but have omitted these from the graphical representation to keep the figure clear.

Apart from our central variables of interest, we include additional variables that have been evidenced to impact attitudes toward immigration and asylum. In line with previous research, we expect more religious individuals to be more accepting of immigrants and asylum seekers (Lubbers et al., 2006; Bohman and Hjerm, 2014; Davidov et al., 2014). Although religious individuals might be more conservative, they seem simultaneously more inclined to be willing to aid others generally and asylum seekers specifically (Lubbers et al., 2006). To control—at least partially—for regional differences within countries, we also include the impact of living in an urbanized or a rural region. While ethnic competition might be larger in cities, there is at the same time more potential for intergroup contact, which might foster more open attitudes (Schneider, 2008). As a last predictor, we focus on the role of perceptions of unsafety in one's neighborhood. Individuals who feel unsafe in their neighborhood and attribute this to immigrants or asylum seekers can develop more negative attitudes (Rustenbach, 2010).

## Contextual Differences and Attitudes Toward Asylum Policy

Although previous studies have tried to unravel contextual effects on support for open vs. restrictive asylum policies across Europe (Coenders et al., 2004; Ivarsflaten, 2005), these studies were mainly conducted in light of the previous asylum “crisis” in the early 2000s. Yet, the current situation is profoundly different. During the aftermath of the Kosovo War in the early 2000s, migration movements were relatively gradual, intra-European and believed to be mostly temporary. The current peak of asylum applications conversely includes more rapid, large and long-term extra-European migration flows (Heisbourg, 2015). Moreover, countries now deal with applications from a wider variety of countries of origin, which increases the duration of application processing and, consequently, pressures asylum systems (OECD, 2015, p. 7). This new context has widened the gap between the opposing political perspectives on the asylum “crisis” as well as between different European states (Castells, 2018; Triandafyllidou, 2018; Zaun, 2018). The varying national circumstances across Europe raise concerns that civil society is becoming equally polarized in camps contra and pro the introduction of open asylum policies. To date, we do not know how exactly the diverging national contexts affect European citizens' attitudes toward asylum policy and whether they complicate a common European response to the renewed inflow of asylum seekers.

In studying how contextual factors relate to asylum policy preferences, we focus on determinants that are of particular importance in current policy debates and oppositions within Europe. Arguments related to structural barriers such as the number of asylum seekers already taken in, the difficulty of integrating various ethnic groups or the economic burden this poses, have been especially prevalent in discussions on the intake of asylum seekers and refugees (e.g., Funk, 2016; Bucken-Knapp, 2017; De Cleen et al., 2017). Moreover, we aim to uncover whether the unique and relatively new context has deteriorated public opinion toward states' policies on asylum and migration. While we recognize that many other indicators pertaining to the general social climate, such as political and media discourses, could be highly influential, this study is restricted to the analysis of the impact of some objective country-level indicators of the policy, economic and migratory context as a necessary first step in the understanding of attitudes toward asylum policy.

As an indicator of policy context, we examine whether the actual generosity of asylum policies is related to the preferred openness vs. restrictiveness of asylum policies. In this regard, we use the rate of positive decisions on asylum applications in a given country (i.e., recognition rate). This indicator is relevant because, in contrast to other more generic policy indicators (e.g., Migration Integration Policy Index scores), it is an indicator of the actual pursued asylum policy rather than of more general integration or immigration approaches. The recognition rate is closely connected to the composition of asylum applicants in terms of countries of origin, as countries receiving many applicants from clear conflict regions (e.g., Syria or Afghanistan) should be more likely to take positive decisions. We nevertheless partly control for the differentiation in profiles of asylum seekers between countries (see further). Moreover, even if applicants from the same country of origin are compared, there are vast differences between European member states in the proportion of applications they recognize (Berger and Heinemann, 2016). These differences in recognition rates could be related to public opinion in various ways. On the one hand, higher recognition rates could lead to more open attitudes, as they point to a governmental endorsement of the admittance of asylum seekers, which in turn stimulates more welcoming attitudes (Esses et al., 2017). Policies may have a norm-shaping function by institutionalizing norms on the ways to treat asylum seekers and on the desired role of migrants in society (Schlueter et al., 2013). On the other hand, higher recognition rates may also intensify concerns about larger numbers of asylum seekers settling in the country, which might instigate threat perceptions and restrictive preferences. Previous studies suggest that open immigration policies are linked to lower rather than stronger threat perceptions and that the norm-shaping function is most plausible (Schlueter et al., 2013; Green et al., 2019). Consequently, we hypothesize that higher recognition rates will lead to higher support for open policies (*Hypothesis 5*).

The financial and economic crisis have furthered the relevance of economic arguments to legitimize the restriction of rights of asylum seekers (Trauner, 2016). Moreover, economic factors, such as GDP and unemployment rates, constitute an important dimension in the design of initial relocation schemes meant to fairly distribute asylum seekers over member states (Niemann and Zaun, 2018). As economic conditions also influence the intensity of ethnic competition and the degree of solidarity citizens are willing to show (van Oorschot, 2008; Meuleman et al., 2017), they are expected to be relevant not only to explain divergence in the intake of asylum seekers but also to interpret differences in attitudes. We focus specifically on long-term unemployment rates to study how the economic context and scarcity of jobs in the aftermath of the financial crisis of 2008 impact asylum policy preferences. To nevertheless also take into account that changes in unemployment rates rather than absolute levels might impact opinions (Meuleman et al., 2017), we include the impact of the rise in unemployment over 10 years as a robustness check (see Table A4 in appendix). Following group conflict theory, attitudes should be especially restrictive in a situation of high unemployment, as this reinforces labor market competition (Scheepers et al., 2002; Meuleman et al., 2017). Furthermore, higher unemployment rates might instigate concerns about the financial implications of accommodating and integrating asylum seekers, thus stimulating preferences for more restrictive asylum policies (*Hypothesis 6*).

As we expect that the number of asylum seekers and the countries of applicants' origin can reflect differently on the attitudes toward asylum policy, two dimensions of the migratory context are taken into consideration. Disentangling these two dimensions offers a more precise understanding of how the so-called “asylum crisis” and arguments uttered in policy debates on the numbers and types of asylum seekers coming in reflect on public opinions. First, the number of asylum applicants in 2016 is included, as a larger number of asylum applications in a country (that is, outgroup size) may increase the saliency of migration issues and the actual competition over material and cultural resources. Considering the number of asylum applications over 2015 and 2016 together might be also relevant. For that reason, the relationship with the average number of applicants over both years is also tested (see Table A4 in appendix). According to GCT, competition induces threat perceptions and negative attitudes toward asylum seekers (Scheepers et al., 2002; Zaun, 2018). However, simultaneously a larger outgroup size increases the probability of intergroup contact, which promotes more open attitudes (Schneider, 2008; Schlueter and Scheepers, 2010). Contact may nevertheless be relatively limited with asylum applicants, as they are often retained in centers that allow relatively limited contact with majority populations. The number of asylum seekers coming in might thus stimulate public reactions of threat more than intergroup contact. Hence, we expect higher support for restrictive policies in countries facing higher levels of asylum applications (*Hypothesis 7*). To also include the impact of the size of outgroups in broader terms and to contrast the threat with the contact hypothesis, we test the impact of the percentage of the foreign-born population from outside the EU as a robustness check (see Table A4 in appendix). Second, asylum seekers from Middle Eastern conflict regions are mostly seen as the “real refugees” and are considered to be more deserving than, for instance, African refugees (Holmes and Castañeda, 2016). As these asylum seekers are considered more deserving of help, higher shares of applications from these regions might strengthen the credibility of the humanitarian frame and stimulate empathy with asylum seekers and their search for protection. The higher the percentage of asylum applications from these regions, the higher the support for open policies is expected to be (*Hypothesis 8*).

## Data and Measurement

### Data

We use data of 2016 from the European Social Survey (ESS) round 8 for the analyses (*dataset version 2.0; doi: 10.21338/NSD-ESS8-2016*) that includes multiple measures of attitudes toward asylum policy. The ESS consists of probability-based samples of the resident population of over 15 years of age that are interviewed by means of face-to-face surveys. Attitudes toward asylum policy are investigated in 20 European countries, namely: Austria, Belgium, Czech Republic, Estonia, Finland, France, Germany, Iceland, Italy, Ireland, Lithuania, the Netherlands, Norway, Poland, Portugal, Slovenia, Spain, Sweden, Switzerland and the United Kingdom (see Table A1 in appendix for sample sizes and descriptive statistics). Originally 23 countries participated in the eighth round of the ESS, but Hungary, Israel and Russia are not included in the analysis. While this is related to the exclusion of one of the items on asylum policy in the questionnaire of Hungary, Russia and Israel are omitted because they do not constitute European countries. Foreign-born respondents, individuals who are not a citizen of the given country and individuals who indicate that they are part of a minority group are excluded from the analysis, as attitudes of the majority group are investigated. The total sample is equal to 32,705. To understand attitudes toward asylum policy in light of the current context, data of ESS round 1 collected in the aftermath of the previous “asylum crisis” (2002/2003) are also used as a point of comparison.

### Indicators

#### Dependent Variable

Attitudes toward asylum policy are measured by two items on a 5-point scale (agree strongly-disagree strongly). The items refer to the extent to which the government should be generous in judging asylum applications (*gvrfgap*) and to whether refugees should be allowed to bring family members (*rfgbfml*). There is also a third item (*rfgfrpc*) in the ESS, which probes whether or not respondents believe that refugee applicants are in real fear of persecution in their country of origin. Because the item loads relatively poorly on the shared latent variable and refers to more general attitudes toward asylum seekers instead of asylum policies, it is, however, omitted from the analyses. The inclusion of only two items is of course not optimal, as it decreases the degrees of freedom and complicates the evaluation of the fit of the measurement model. As the same items are used to operationalize the latent concept on the individual- and the country-level, a two-level confirmatory factor analysis is applied to assess to validity of the concept on the within—as well as on the between-level. Factor loadings are set equal across levels to guarantee that the construct can be interpreted similarly at the individual—and country-level (Tay et al., 2014; Ruelens et al., 2018). The factor analysis shows that factor loadings, which are displayed in Table A2 in appendix together with the question wordings, are sufficiently large on both levels. Higher scores on the latent variable indicate more support for restrictive policies. Immigration policy preferences are also included as a dependent variable in appendix to check the differences with asylum policy preferences. This is operationalized through three four-point items asking to what extent immigration should be allowed or restricted (*imsmetn, imdfetn*, and *impcntr*).

#### Individual Independent Variables

Economic (*imbgeco*) as well as cultural threat (*imueclt*) are each measured by one item that assesses whether respondents believe that immigrants are good or bad for the economy and cultural life, respectively (11-point scale). Both items are reversed so that higher scores indicate higher levels of ethnic threat. The human values are measured by means of the shortened version of the Portrait Value Questionnaire (PVQ, Schwartz et al., 2001) included in the ESS. Respondents are asked to what extent they identify with given hypothetical portraits of people (6-point answer scale). For the unified value conformity-tradition the four portraits encompass: showing modesty (*ipmodst*), attaching importance to tradition (*imptrad*), behaving properly (*ipbhprp*), and doing what you are told (*ipfrule*). The three items of universalism measure the importance of: listening to different people (*ipudrst*), treating everyone equally (*ipeqopt*) and caring for nature and the environment (*impenv*). The items are all reversed so that higher scores indicate higher identification with the two human values. The validity of the two constructs is assessed by means of confirmatory factor analysis (see Table A3 in appendix for more details). All factor loadings are sufficiently large and, after implementing two theoretically justified error correlations, the fit of the model is adequate according to most criteria (*X*^2^ = 1310.374; *df* = 11; CFI = 0.945; RMSEA = 0.060; SRMR = 0.036). The TLI (0.896) is on the low side, but modification indices show that the model does not contain substantial local misfit.

As indicators of socio-economic status, we use occupation, education as well as subjective income. Occupation is divided by the Erikson-Goldthorpe-Portocarero scheme (Ganzeboom and Treiman, 1996) into six classes: the service class, white collar workers, blue collar workers (reference category), the self-employed, the unemployed, and the retired and other non-actives. Dividing occupation into EGP-based classes allows to offer more detailed insight into how various groups view asylum policies and goes further than generic and one-dimensional measurements of social status. Educational attainment (*edulvlb*) is measured through ISCED classification, which is used to create three categories: lower (secondary) education, higher secondary education (reference category), and tertiary education. To indicate their subjective income (*hincfel*), which has far less missing values than objective measures (0.8 vs. 16%), respondents were offered the following four categories: “living comfortably on present income,” “coping on present income,” “finding it difficult on present income,” and “finding it very difficult on present income.” This categorical variable is also divided into several dummy variables to provide more insight into whether each of the categories score differently on support for restrictive asylum policies. The following variables are also included in the statistical models: age, gender, religiosity (*rlgdgr*; 0–10), perceived safety in one's neighborhood (*aesfdrk*; recoded so that 1 = safe, 0 = not safe) and area of residence (*domicil*; recoded so that 0 = rural area, 1 = big city, suburbs or town).

#### Contextual Independent Variables

Four contextual variables are included: the recognition rate of asylum applications, the long-term unemployment rate, the number of asylum applications per 1,000 inhabitants and the share of these applications from Syria, Afghanistan and Iraq. Based on Eurostat data (2016b), the recognition rate of applications for asylum is calculated by dividing the number of positive decisions (granting refugee status) by the total number of decisions taken in a given country. For unemployment rates, the average long-term unemployment rate over the years of 2011 to 2016 is taken from the World Development Indicators database of the World Bank (The World Bank, 2016). Taking the average guarantees that the used unemployment rates represent a more stable figure. As mentioned, we compare the result of this long-term unemployment rate to the impact of the change in unemployment rate between 2006 and 2016 (see Table A4 in appendix). To calculate the number of asylum applications per 1,000 inhabitants, data from Eurostat on the number of asylum applications in 2016 as well as on the size of the native population in 2016 are used (Eurostat, 2016a,c). As a robustness check, we also check the impact of the number of asylum seekers per 1,000 inhabitants averaged over 2015 and 2016. The role of the percentage of the population born outside the EU is also considered as an alternative operationalization of the size of the outgroup (Eurostat, 2016c). The share of applications from Middle Eastern conflict regions is obtained by dividing the number of applications of individuals from Syria, Iraq, and Afghanistan by the total number of applications in 2016 (Eurostat, 2016a). We focus on the share of asylum applications from these countries of origins, as they constituted some of the most evident conflict regions and encompassed the largest represented nationalities among applicants in 2016 (UNHCR-The UN Refugee Agency, 2017). Applicants from these countries are, as a result, most likely to be considered credible candidates for the acquirement of refugee status. The scores on these contextual variables per country are displayed in Table A1 in appendix.

### Statistical Modeling

**Figure 2:**
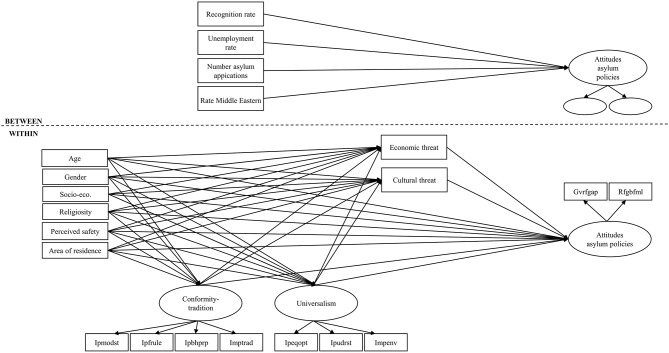
Model with estimated empirical relationships.

To estimate all the relationships on the individual- as well as on the country-level and to take the clustered nature of the data into account, multilevel structural equation modeling is applied (Meuleman, 2019). This SEM-based approach has the advantage to make it possible to model measurement error and to specify indirect effects. The choice for multilevel analysis is supported by the fact that no less than 20.7% of the variance of attitudes toward asylum policy is located at the country level. The estimated structural model with all included variables and empirical relationships is visualized in Figure 2. Note that the individual-level predictors are only included at the within level (the between-level components are thus omitted). The reason is that the small sample size at the higher level limits the complexity of the between-level model that can be estimated. In order to remediate the effects of the limited number of higher-level units (20 countries), we use a Bayesian estimator that improves accuracy of the results (Meuleman and Billiet, 2009; Hox et al., 2012; Stegmueller, 2013). For the Bayesian estimation the Gelman-Rubin criterion is used to examine convergence (the cut-off value is set to 0.01) and two chains of the Gibbs sampler are requested (Gelman et al., 2004; Hox et al., 2012). The number of iterations is set to 10,000 to facilitate convergence and a thinning factor of 50 is used to counter autocorrelation (Muthén, 2010). Trace and autocorrelation plots are also inspected to assess convergence and the absence of autocorrelation. As Bayesian estimation provides little information on the fit of the model, indices are obtained by re-estimating the models with a robust maximum likelihood estimation procedure.

All analyses are conducted with Mplus version 7.3 (Muthén and Muthén, 2012). To deal with item non-response, the Full Information Maximum Likelihood (FIML) procedure is used. Cases with missings are included in the calculation of pairwise correlations, unless they have missing values on all endogenous variables or on one of the exogenous variables. This is the case for 1,109 cases (3.4%), resulting in a final sample size of 31,596. The syntax for data preparation in SPSS and for the statistical modeling in Mplus can be found as a Supplementary File (Data Sheet 1)

## Results

### Descriptive Overview: Comparing Attitudes Toward Asylum Policies Between 2002 and 2016

**Figure 3:**
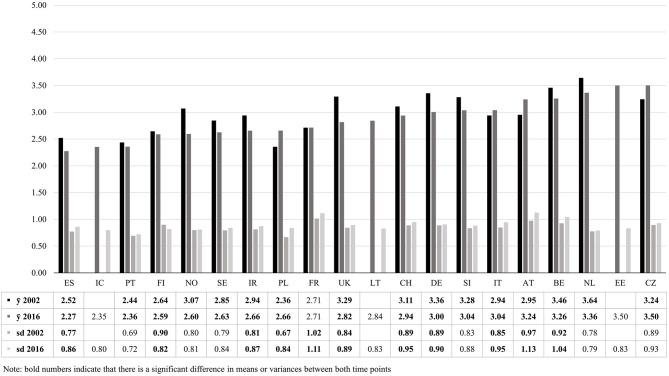
Means (ȳ) and standard deviations (sd) of attitudes toward asylum policy in 2002 and 2016.

To gain insight in how the contemporary context of the “asylum crisis” bears on public opinion, we start by comparing attitudes toward asylum policy between 2002 and 2016 (see Figure 3). The point of comparison (2002) is the aftermath of the Kosovo War. Besides country means, we also compare a measure of dispersions (the standard deviation). This allows us not only to see whether support for restrictive asylum policies has evolved, but also to investigate whether an increase in polarization of the public opinions on asylum policy can be witnessed.

Interestingly, Figure 3 indicates that there is considerable geographical variation in the support for the restrictiveness of policies, as means for 2016 range from 2.27 in Spain to 3.50 in the Czech Republic (higher values represent more support for restrictive policies). Citizens in the Southern and Nordic countries are generally the most in favor of open asylum policies, while the strongest support for restrictive policies can be witnessed in Central- and West-European countries. Some of the countries that received the lowest number of applications (e.g., Czech Republic and Estonia) are simultaneously characterized by restrictive opinion climates. This pattern illustrates that the countries who face the highest number of applications are not necessarily most restrictive in their attitudes (see Table A1 in appendix for numbers of asylum applications per country). Public opinion divides do not necessarily mirror differences in the intake of asylum seekers across Europe.

The over-time comparison teaches us that attitudes have become significantly more favorable between 2002 and 2016 in twelve countries (out of the 17 with available data for both rounds)^1^. Only in Poland, Italy, Austria and the Czech Republic have attitudes become significantly more restrictive. As Poland and the Czech Republic have not faced high numbers of applications over the recent years, this again illustrates negative sentiments cannot be traced back to heightened inflows of asylum seekers. Although attitudes have become more open in most countries, Figure 3 also shows that the dispersion of opinions has increased between 2002 and 2016 in many countries. In nine countries, we see a significant increase of the standard deviation of attitudes toward asylum policy, while a significant decrease is only found in one country. The slightly increasing support for open policies thus coincides with more diversified attitudes toward asylum policy. This may point to a growing polarization within countries and a deepening cleavage between the humanitarian and exclusionary perspectives on asylum.

Although the most restrictive attitudes and an increase in negative opinions are not to be found in countries facing the highest numbers of asylum applications, the dividing lines between countries are not random either. Restrictive attitudes are especially prevalent in countries where the issue of migration has been heavily politicized. Countries with an open opinion climate, such as Portugal and Spain, are characterized by low migration salience and the absence of strong anti-immigration parties (Dennison and Geddes, 2019). Countries with higher and growing support for restrictive policies such as Austria experienced increasing negative politicizations of the asylum issue (Gruber, 2017). It is no coincidence that two of the four countries where attitudes became more restrictive over time (i.e., Czech Republic and Poland) are part of the Viségrad states, which have been most vocal in opposing open policies and the introduction of a common quota system (Zaun, 2018). Consequently, rather than mirroring divides in numbers of asylum applicants between European countries, attitudes toward asylum policy seem susceptible to the politicization of the asylum issue.

### Individual and Contextual Determinants of Attitudes Toward Asylum Policy

To gain more insight into the individual and contextual characteristics that shape attitudes toward asylum policy, we estimate a multilevel structural equation model^2^ (see visualization in Figure 2). Hereby we focus on the most recent ESS round of 2016. Table 1 displays standardized regression coefficients (and 95% posterior probability intervals) obtained by Bayesian estimation^3^. The effects are discussed following the order of the presumed causal chain of the model.

**Table 1:** Standardized parameter estimates and posterior probability intervals of a model predicting attitudes toward asylum policy (*N* = 31,596).

	**Conformity-tradition**		**Universalism**		**Cultural threat**		**Economic threat**		**Attitudes toward asylum policy**	
	**Estim**.	**95% PPI**	**Estim**.	**95% PPI**	**Estim**.	**95% PPI**	**Estim**.	**95% PPI**	**Estim**.	**95% PPI**
**INDIVIDUAL VARIABLES**										
**Gender**										
Female (ref)										
Male	0.026^*^	[0.013 to 0.040]	−0.085^*^	[−0.099 to −0.069]	0.001	[−0.011 to 0.012]	−0.054^*^	[−0.065 to −0.043]	0.037^*^	[0.024 to 0.051]
**Age**	0.172^*^	[0.158 to 0.186]	0.028^*^	[0.013 to 0.044]	0.004	[−0.008 to 0.017]	−0.018^*^	[−0.030 to −0.005]	0.018^*^	[0.004 to 0.032]
**Education**										
Lower (secondary)	0.022^*^	[0.008 to 0.038]	−0.027^*^	[−0.043 to −0.011]	0.000	[−0.012 to 0.013]	0.029^*^	[0.017 to 0.042]	−0.044^*^	[−0.059 to −0.030]
Higher secondary (ref)										
Tertiary	−0.075^*^	[−0.091 to −0.060]	0.098^*^	[0.082 to 0.114]	−0.096^*^	[−0.109 to −0.083]	−0.089^*^	[−0.102 to −0.076]	−0.009	[−0.024 to 0.006]
**Subjective income**										
Comfortable (ref)										
Coping	0.018^*^	[0.003 to 0.033]	−0.076^*^	[−0.092 to −0.060]	0.061^*^	[0.048 to 0.073]	0.063^*^	[0.051 to 0.075]	−0.024^*^	[−0.038 to −0.011]
Difficult	0.010	[−0.005 to 0.025]	−0.065^*^	[−0.081 to −0.048]	0.105^*^	[0.093 to 0.117]	0.114^*^	[0.102 to 0.126]	0.000	[−0.015 to 0.014]
Very difficult	−0.001	[−0.015 to 0.014]	−0.044^*^	[−0.059 to −0.029]	0.085^*^	[0.074 to 0.097]	0.090^*^	[0.079 to 0.102]	0.027^*^	[0.014 to 0.042]
**Occupation**										
Service	−0.001	[−0.017 to 0.014]	0.027^*^	[0.010 to 0.043]	−0.028^*^	[−0.041 to −0.016]	−0.032^*^	[−0.045 to −0.020]	−0.008	[−0.023 to 0.006]
Blue collar (ref)										
White collar	0.000	[−0.020 to 0.020]	0.053^*^	[0.032 to 0.073]	−0.050^*^	[−0.066 to −0.034]	−0.058^*^	[−0.075 to −0.042]	−0.004	[−0.023 to 0.014]
Self–employed	−0.015	[−0.031 to 0.000]	0.038^*^	[0.021 to 0.055]	−0.017^*^	[−0.030 to −0.003]	−0.029^*^	[−0.042 to −0.016]	0.012	[−0.002 to 0.027]
Unemployed	−0.014	[−0.029 to 0.001]	0.057^*^	[0.040 to 0.073]	−0.010	[−0.023 to 0.002]	0.008	[−0.004 to 0.021]	−0.014^*^	[−0.028 to 0.000]
Retired/non–active	0.006	[−0.014 to 0.027]	0.086^*^	[0.063 to 0.107]	−0.068^*^	[−0.086 to −0.051]	−0.072^*^	[−0.089 to −0.055]	−0.057^*^	[−0.076 to −0.037]
**Religiosity**	0.290^*^	[0.276 to 0.304]	0.082^*^	[0.067 to 0.096]	−0.098^*^	[−0.110 to −0.085]	−0.102^*^	[−0.115 to −0.090]	−0.047^*^	[−0.062 to −0.033]
**Area of residence**										
Rural area (ref)										
Big city, suburbs or town	−0.061^*^	[−0.074 to −0.047]	0.014	[0.000 to 0.028]	−0.038^*^	[−0.049 to −0.027]	−0.055^*^	[−0.066 to −0.044]	−0.011	[−0.024 to 0.001]
**Perceived safety**										
Not safe (ref)										
Safe	−0.046^*^	[−0.060 to 0.032]	0.063^*^	[0.048 to 0.078]	−0.103^*^	[−0.115 to −0.092]	−0.089^*^	[−0.100 to −0.078]	−0.024^*^	[−0.038 to −0.011]
**Conformity–tradition**					0.392^*^	[0.372 to 0.413]	0.325^*^	[0.304 to 0.345]	0.168^*^	[0.142 to 0.194]
**Universalism**					−0.412^*^	[−0.431 to −0.393]	−0.328^*^	[−0.347 to −0.310]	−0.286^*^	[−0.311 to −0.261]
**Economic threat**									0.272^*^	[0.255 to 0.289]
**Cultural threat**									0.328^*^	[0.310 to 0.347]
***R***^**2**^ **(within)**	0.169		0.050		0.261		0.205		0.497	
**CONTEXTUAL VARIABLES**										
**Number asylum applications**									0.001	[−0.444 to 0.444]
**% conflict regions**									−0.171	[−0.703 to 0.412]
**% unemployment**									−0.581^*^	[−0.914 to −0.114]
**Recognition rate**									0.382	[−0.201 to 0.861]
***R***^**2**^ **(between)**									0.497	

PPI, posterior probability interval;

* *P < 0.05; X^2^ = 1847.645; df = 128; CFI = 0.919; TLI = 0.854; RMSEA = 0.021; SRMR_W_ = 0.024; SRMR_B_ = 0.075*.

Table 1 shows that, on the individual-level, men, older respondents, lower-educated individuals, respondents coping with their present income, more religious individuals, those from rural areas and who feel unsafe underscore the value conformity-tradition the most. This might be because these groups rely more on traditional ties and habitual patterns (Schwartz, 2007). In contrast, women, older respondents, higher-educated individuals, individuals living comfortable on their income, respondents that are not blue-collar workers, those who are more religious and those that feel safe put greater emphasis on the value of universalism. The higher scores on universalism of individuals with a higher socio-economic status are not surprising, as education stimulates openness and a higher income facilitates a broader worldview (Schwartz, 2007).

Individuals who emphasize the value of universalism, in turn, feel economically and especially culturally less threatened. Universalism includes a transcendence of individual interests and individuals that stress its importance are, consequently, less inclined to perceive a clash in interests and to feel threatened in their well-being (Meuleman and Billiet, 2018). The value of conformity-tradition conversely stimulates perceptions of economic and cultural threat, which can be understood from a stronger fear for deviant norms and alternative beliefs (Davidov et al., 2014). In addition, individuals who have not completed tertiary education, with a lower subjective income, blue collar workers, less religious respondents, individuals from rural areas, and those who feel unsafe perceive stronger economic and cultural threat. Rather surprisingly, unemployed respondents do not feel economically more threatened, but this might be related to the larger fear of becoming unemployed due to immigration among employed respondents (and especially blue-collar workers) (O'Rourke and Sinnott, 2006). In addition, results indicate that the lower educated do not directly feel more culturally threatened. This might be because the relationship is mainly indirect (through conformity-tradition) rather than direct. In general, respondents from deprived backgrounds feel indirectly, through their higher identification with conformity-tradition and lower emphasis on universalism, as well as directly more threatened.

Consistent with hypotheses 1 and 2, economic and cultural threat foster support for restrictive asylum policies. The sizes of the regression coefficients of both economic threat (*b* = 0.27) and cultural threat (b = 0.33) are quite large, which points to the importance of socio-tropic concerns about the economic and cultural impact in shaping attitudes. Those who adopt negative frames and, as a result, believe migration damages the economic situation and erodes cultural unity, advocate for stricter policies in order to protect the welfare and traditional norms of their group (Ivarsflaten, 2005). Although economic and cultural threat perceptions thus correspond to distinct frames on the asylum issue, they have a similar impact on attitudes toward asylum policy. Apart from the influence of threat perceptions, the two human values directly and indirectly influence support for restrictive policies. In line with hypothesis 3, universalism stimulates the promotion of open policies, which might be due to its coincidence with a humanitarian framework that focuses on the needs of asylum seekers and on helping others more generally (Tartakovsky and Walsh, 2016; Ritter and Rhomberg, 2017). Conformity-tradition, in contrast, fuels reluctance to admit asylum seekers. This lends support to hypothesis 4 and is related to the overlap of conformity-tradition with discourses that portrays asylum seekers as damaging to the social cohesion and existing normative order (Tartakovsky and Walsh, 2016). Together all of these individual-level variables are able to explain almost half of the within-variance of attitudes toward asylum policies. Clearly, the individual-level mechanisms included in the model have high explanatory power.

On the contextual-level, results indicate that most of our economic, policy, and migratory indicators are not capable of explaining cross-national differences in attitudes toward asylum policy. Although there is considerable variation in recognition rates between countries—as indicator of the pursued approaches in handling the increase in asylum applications (Berger and Heinemann, 2016)—this does not reflect on attitudes toward asylum policy among the population at large. The standardized coefficient for recognition rate is relatively large and positive (*b* = 0.38), but insignificant. This refutes hypothesis 5. The two dimensions of the migratory context do not significantly impact attitudes either. The number of asylum applications in a given country cannot account for differences in opinion climates between European states (*b* = 0.00). While this is in contrast to hypothesis 7 and common expectations (e.g., Zaun, 2018), the null effect of the size of the outgroup confirms the observed patterns in the descriptive overview. The countries that have taken in most asylum seekers are not necessarily confronted with a backlash in public opinion. The wide variation in the countries of origin of the incoming asylum seekers does not seem to matter for public preferences either: The share of asylum seekers from Middle Eastern conflict regions is not significantly related to public attitudes. There is thus no support for hypothesis 8, which states that higher shares of these groups would stimulate support for more open policies.

**Figure 4:**
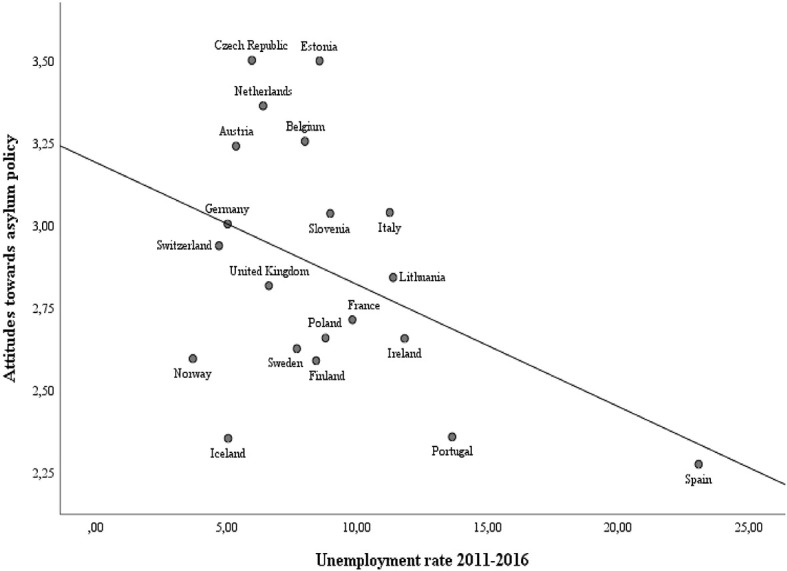
Scatterplot of country means of attitudes toward asylum policy and average unemployment rates between 2011 and 2016.

The long-term unemployment rate is the only contextual indicator that is significantly related to attitudes toward asylum policy. Contrary to hypothesis 6, the influence of the unemployment rate is negative rather than positive, which would mean that stronger support for open asylum policies is precisely found in contexts with higher unemployment rates (and thus more adverse economic conditions). However, when plotting the relationship between average attitudes toward asylum policy and long-term unemployment rates (see Figure 4), it becomes evident that the significant regression coefficient of unemployment rate is mainly attributable to the cases of Spain and Portugal. Both countries are characterized by high average support for open policies as well as high unemployment rates (13.63% in Portugal and 23.07% in Spain), which heavily impacts the relationship. Several other countries combine low unemployment rates with restrictive preferences, which is in line with the direction of the relationship, yet this pattern does not hold across the whole set of countries under investigation. In addition, the regression coefficient of the long-term unemployment rate becomes insignificant when estimating the same structural model without Spain. This confirms that the effect is mainly driven by one influential case and that the significant coefficient of long-term unemployment rates thus needs to be qualified. In any case, these results disconfirm the competition hypothesis stating that economic recessions fuel conflicts over material resources (Scheepers et al., 2002; Meuleman et al., 2017).

The robustness checks with alternative operationalization of the migratory and economic context (extra-EU foreign-born population, the number of asylum applicants averaged over 2015 and 2015, and the change in unemployment rates between 2006 and 2016) also indicate that these contexts do not have an important influence (see Table A4 in appendix). The results do not change substantially when using these different indicators, which strengthens our main conclusions. Our paper demonstrates that strong divides in attitudes toward asylum policy exist between the European populations under analysis, but that the contextual indicators do not explain these country-level differences. Neither the policy context, nor the economic or migratory context are able to uncover the nature of the divisions in opinion climates. Some of the factors that have been suggested as causes of citizens' opposition against the arrival of asylum seekers, such as the unemployment rate and the number of asylum seekers entering (Niemann and Zaun, 2018), are not systematically related to the public opinion climate.

### Comparing Attitudes Toward Asylum Policies and Attitudes Toward Immigrant Policies

When attitudes toward immigration policy in general are analyzed instead of attitudes toward asylum policy, we reach quite similar conclusions, and roughly the same set of predictors is relevant (see Table A5 in appendix). Similarly to attitudes toward asylum policy, economic, and cultural threat perceptions foster support for curbing immigration. The two human values also have an equivalent impact, with conformity-tradition leading to more restrictive attitudes and universalism stimulating open views. Those with a lower socio-economic status are also both directly and indirectly more opposed to allowing immigrants entry into the country. In addition, the four contextual predictors are not significantly related to the public opinion climate, which also confirms the conclusion that structural factors are not decisive in shaping the attitudes of the public. There are nevertheless also some differences with attitudes toward asylum policy. While for attitudes toward asylum policy the cultural threat variable has the strongest impact, for immigration policy preferences perceptions of economic threat are more important. This indicates that cultural arguments are more relevant in discussions on asylum and economic discourses more influential in debates on immigration in general. The stronger impact of the cultural threat variable might, however, be specific to the current context, as many incoming asylum applicants have non-European backgrounds. In addition, the explained variance on the context-level is much lower for opinions on immigration policy. These results indicate that although it is theoretically relevant to distinguish between attitudes toward various migrant groups, empirically the same structural drivers are at play in shaping these opinions. This seems to indicate that restrictive attitudes toward different migrant groups often occur together and that opinions on specific groups are actually an expression of a diffuse attitude toward ethnic outgroups in general (Meuleman and Billiet, 2003).

## Conclusion

In light of the current context, characterized by increased inflows of asylum seekers as well as deepening European cleavages in perspectives on appropriate political responses, this paper set out to gain deeper insight into majority-group members' attitudes toward open vs. restrictive asylum policies within and between European societies. We realized this by focusing on three aspects: (1) a descriptive overview of how attitudes toward asylum policy varied across European countries between 2002 and 2016; (2) an analysis of the impact of individual-level factors connected to dominant arguments and frames used in asylum debates, with a specific focus on threat perceptions and human values; (3) an examination of how the structural factors referred to in public debates on asylum explain differences between member states in opinions on policies, with particular attention for the influence of the policy, economic and migratory context.

A first finding is that between 2002 and 2016 support for open policies has become stronger in a majority of countries. Although the current situation differs profoundly from the context at the beginning of the century in terms of diversity and pace of the inflow of asylum seekers (Heisbourg, 2015; OECD, 2015), in most countries citizens are now more supportive of generous admission policies than in 2002. Nevertheless, we did find strong regional variations in attitudes toward asylum policy as well as growing polarizations within European countries. In line with the growing divergence in terms of political responses to the crisis as well as in discourses being adopted (Ritter and Rhomberg, 2017; Castells, 2018; Triandafyllidou, 2018), public opinions on the European continent tend to become more divided. While in the Nordic and Southern countries the public seems to favor open polices that admit larger quantities of asylum seekers, attitudes in several Western- and Central-European countries are far more restrictive. The restrictive opinion climates in countries such as the Czech Republic and Estonia cannot be understood on the basis of GCT, as these countries received a negligible share of the total asylum seekers in Europe and the Czech Republic was one of the countries that did not relocate any asylum seekers (Niemann and Zaun, 2018; Zaun, 2018). Countries who faced the largest numbers of asylum applications are not necessarily the ones where the public opinion climate is most negative.

On the individual level, discourses appeared to be relevant, as we found a strong impact of several individual factors that are closely related to dominant discourses on asylum. In accordance with frames or discourses that portray refugees as economically burdensome and as culturally deviant (Greussing and Boomgaarden, 2017; Ritter and Rhomberg, 2017), economic and cultural threat perceptions fostered restrictive attitudes. Socio-tropic concerns about the impact of the inflow of immigrants on the economy and cultural life appeared to be of great importance in shaping attitudes. Apart from threat perceptions, the two human values universalism and conformity-tradition had a considerable impact. Universalism, which coincides with a humanitarian frame (Tartakovsky and Walsh, 2016), led to weaker concerns about the impact of migration and to more support for open policies. Equivalent to concerns for the preservation of the Western liberal core values in political debates (Lucassen, 2018), conformity-tradition fuelled economic and cultural fears, and opposition to open migration policies.

Another finding is that our indicators of policy and migratory context are unable to explain the cross-national attitudinal differences. Differences in cross-national circumstances that were given a lot of weight in national and European debates on how to approach and distribute the rise in asylum applications, such as the number of asylum seekers and the economic contexts (Trauner, 2016; Niemann and Zaun, 2018), seem to be not at all related to the asylum or immigration policies citizens prefer. Although a significant negative effect of the long-term unemployment rate was found, this relationship was mainly driven by the specific positions of Spain and Portugal. Our results illustrate that contrarily to the GCT-based claim that adverse economic contexts and larger sizes of the outgroup would instigate anti-migrant sentiments, the actual competitive and policy contexts are incapable of predicting country divisions in attitudes toward asylum policy.

The absence of effects of the migratory and economic context suggests that other factors might be more relevant to understand diverging attitudes toward asylum policy across European countries. Although we did not test this explicitly, our results suggest that public opinion mirrors dividing lines in dominant political perspectives and discourses. The rather restrictive opinion climate of the Central-European countries, for instance, resembles the strong resistance of policy makers in these countries (including the Visegrád group) against open policies and the adoption of quota (Veebel and Markus, 2015; Niemann and Zaun, 2018; Zaun, 2018). Previous research has indeed evidenced that media framings and political discourses may influence anti-immigrant attitudes (Bos et al., 2016; Matthes and Schmuck, 2017; Czymara, 2019), while the presence of populist entrepreneurs might also explain more restrictive attitudes toward immigrants in some countries (Wirz et al., 2018).

Our contextual analysis contends with several limitations, however. First, the relatively small number of countries included in the study complicates the application of multilevel models, and even the Bayesian estimation procedure cannot prevent that the tests for context effects have limited power only. The absence of significant effects should thus be interpreted with the necessary caution. Second, the included indicators to operationalize these various contexts are, due to limited data availability, not always as optimal as we would like them to be. The recognition rate, for instance, is intertwined with the composition of the backgrounds of asylum applicants, as higher recognitions might occur in countries that have higher shares of applicants from evident conflict regions. In addition, the indicators of the migratory context only consider the asylum applications in a given year and, as a result, fail to adopt a dynamic approach (e.g., Meuleman et al., 2009) as well as to fully grasp the total size and diversity of the outgroup. To address this shortcoming, we used alternative operationalizations of the economic and migratory context as robustness checks, which yielded the same conclusions. This gives additional evidence that structural factors do not really seem to strongly determine attitudes toward asylum policies. As the symbolic discourses being pushed and used as frames of references seem to reflect much harder on the policies deemed appropriate by citizens, indicators more closely related to these popular arguments should thus instead be included. Future research would benefit from a detailed examination of how contextual discourses are reflected in public opinions toward immigrants.

The seemingly higher relevance of political mobilizations and media discourses in understanding attitudes compared to the actual cross-national circumstances also has other implications. The diverging national contexts across EU member states do not seem to negate the development of a common public response to the challenges that the increased inflow of asylum seekers introduces. Contrarily to what is often believed and argued, the differential national contexts as such do not seem to make wide public support for a strong common European asylum system impossible. Instead populist governmental mobilizations and vast differences in adopted discourses might complicate wide public support for shared and open solutions across Europe (Zaun, 2018). As the cases of Hungary and Poland clearly indicate, aggressive mobilizations and strong anti-immigrant rhetoric might instigate drastic increases in anti-migrant sentiments and, as a result, erode the social basis for open and common migration policies (Zaun, 2018; Van Hootegem and Meuleman, 2019).

## Data Availability Statement

The datasets analyzed for this study can be found on the website of the European Social Survey [https://www.europeansocialsurvey.org/data/round-index.html].

## Ethics Statement

Ethical review and approval was not required for the study on human participants in accordance with the local legislation and institutional requirements. Written informed consent for participation was not required for this study in accordance with the national legislation and the institutional requirements.

## Author Contributions

AV designed the study, identified the relevant theoretical frameworks, analyzed the data, and drafted the article. BM designed the study, identified the relevant theoretical frameworks, and corrected the first draft of the article. KA designed the study, identified the relevant theoretical frameworks, and corrected the final draft of the article.

## Conflict of Interest

The authors declare that the research was conducted in the absence of any commercial or financial relationships that could be construed as a potential conflict of interest. The reviewer ES declared a past co-authorship with one of the author BM to the handling editor.

## Footnotes

^1^A set of independent sample t-tests and a number of Levene's tests were conducted to determine whether means and variances are equal across samples.

^2^The estimation of the structural model with a robust maximum likelihood estimator shows that various indices suggest that the fit at the within- and the between-level is adequate (*X*^2^ = 1847.645; *df* = 128; CFI = 0.919; TLI = 0.854; RMSEA = 0.021; SRMR_W_ = 0.024; SRMR_B_ = 0.075). CFI and TLI are too low, which is most likely related to the large number of categorical variables (with a large number of dummies) in the model. These dummy variables typically produce large correlation matrices with many near-zero entries. Such correlation structures typically lead to low incremental fit indices (such as CFI and TLI) because they are based on a comparison with a baseline independence model. Because the observed data structure contains a large number of low correlations, this baseline is not so bad and difficult to improve. The modification indices and the differences between the estimated and observed correlation matrix do not reveal local misfit or plausible alterations that are theoretically defendable and would ameliorate the fit substantially. On the between-level, the elevated SRMR seems to be related to the restriction of equal loadings across levels and the regression of the predictors on the latent concept instead of on the items separately. We nevertheless allow this minor misfit to occur in order to estimate a more parsimonious model that is defendable.

^3^Coefficients are interpretable in a similar fashion as for a regression with a maximum likelihood estimator and the intervals should be interpreted as the 95% probability that the population value falls between the two bounds.
