# Supplementary material for: Attitudes Toward Asylum Policy in a Divided Europe: Diverging Contexts, Diverging Attitudes?
Source: Front Sociol. 2020 May 21;5:35. doi: 10.3389/fsoc.2020.00035 (PMC8022488; doi:10.3389/fsoc.2020.00035)
Supplement: Supplementary file 1 [file Data_Sheet_1.PDF]

SPSS syntax

*Starting dataset: ESS8e02.sav

*CASE SELECTION: deleting Hungarian individuals, individuals born in other country, individuals who are not citizens of country and individuals who identify as being a minority

```
DATASET ACTIVATE DataSet1.
FILTER OFF.
USE ALL.
SELECT IF (cntry ~= "HU" AND brncntr = 1 AND ctzcntr = 1 AND blgetmg = 2).
EXECUTE.
```

*RECODING INDIVIDUAL VARIABLES

*Rescoring the ISCO08 codes to ISCO88 codes to construct the EGP-class typology

```
IF  (isco08 = 1111) iscoco=1110.
if (isco08=1112) iscoco=1120.
if (isco08=1113) iscoco=1130.
if (isco08=1114) iscoco=1141.
if (isco08=1114) iscoco=1142.
if (isco08=1114) iscoco=1143.
if (isco08=1120) iscoco=1210.
if (isco08=1211) iscoco=1231.
if (isco08=1211) iscoco=1317.
if (isco08=1212) iscoco=1232.
if (isco08=1212) iscoco=1317.
if (isco08=1213) iscoco=1229.
if (isco08=1213) iscoco=1239.
if (isco08=1219) iscoco=1227.
if (isco08=1219) iscoco=1228.
if (isco08=1219) iscoco=1229.
if (isco08=1219) iscoco=1231.
if (isco08=1219) iscoco=1317.
if (isco08=1219) iscoco=1318.
if (isco08=1221) iscoco=1233.
if (isco08=1221) iscoco=1317.
if (isco08=1222) iscoco=1234.
if (isco08=1222) iscoco=1317.
if (isco08=1223) iscoco=1237.
if (isco08=1223) iscoco=1319.
if (isco08=1311) iscoco=1221.
if (isco08=1312) iscoco=1221.
if (isco08=1321) iscoco=1222.
if (isco08=1321) iscoco=1312.
if (isco08=1322) iscoco=1222.
if (isco08=1322) iscoco=1312.
if (isco08=1323) iscoco=1223.
if (isco08=1323) iscoco=1313.
if (isco08=1324) iscoco=1226.
```

```
if (isco08=1324) iscoco=1235.
if (isco08=1324) iscoco=1316.
if (isco08=1330) iscoco=1226.
if (isco08=1330) iscoco=1236.
if (isco08=1330) iscoco=1316.
if (isco08=1330) iscoco=1317.
if (isco08=1341) iscoco=1229.
if (isco08=1341) iscoco=1319.
if (isco08=1342) iscoco=1229.
if (isco08=1342) iscoco=1319.
if (isco08=1342) iscoco=2230.
if (isco08=1343) iscoco=1229.
if (isco08=1343) iscoco=1319.
if (isco08=1343) iscoco=2230.
if (isco08=1344) iscoco=1229.
if (isco08=1344) iscoco=1319.
if (isco08=1345) iscoco=1229.
if (isco08=1345) iscoco=1319.
if (isco08=1346) iscoco=1227.
if (isco08=1346) iscoco=1317.
if (isco08=1349) iscoco=1229.
if (isco08=1349) iscoco=1319.
if (isco08=1411) iscoco=1225.
if (isco08=1411) iscoco=1315.
if (isco08=1412) iscoco=1225.
if (isco08=1412) iscoco=1315.
if (isco08=1420) iscoco=1224.
if (isco08=1420) iscoco=1314.
if (isco08=1431) iscoco=1319.
if (isco08=1439) iscoco=1229.
if (isco08=1439) iscoco=1319.
if (isco08=2111) iscoco=2111.
if (isco08=2112) iscoco=2112.
if (isco08=2113) iscoco=2113.
if (isco08=2114) iscoco=2114.
if (isco08=2120) iscoco=2121.
if (isco08=2120) iscoco=2122.
if (isco08=2131) iscoco=2211.
if (isco08=2131) iscoco=2212.
if (isco08=2132) iscoco=2213.
if (isco08=2132) iscoco=3213.
if (isco08=2133) iscoco=2211.
if (isco08=2141) iscoco=2149.
if (isco08=2142) iscoco=2142.
if (isco08=2143) iscoco=2149.
if (isco08=2144) iscoco=2145.
if (isco08=2145) iscoco=2146.
if (isco08=2146) iscoco=2147.
if (isco08=2149) iscoco=2149.
if (isco08=2151) iscoco=2143.
if (isco08=2152) iscoco=2144.
```

```
if (isco08=2153) iscoco=2144.
if (isco08=2161) iscoco=2141.
if (isco08=2162) iscoco=2141.
if (isco08=2163) iscoco=3471.
if (isco08=2164) iscoco=2141.
if (isco08=2165) iscoco=2148.
if (isco08=2166) iscoco=3471.
if (isco08=2211) iscoco=2221.
if (isco08=2212) iscoco=2212.
if (isco08=2212) iscoco=2221.
if (isco08=2221) iscoco=2230.
if (isco08=2222) iscoco=2230.
if (isco08=2230) iscoco=3229.
if (isco08=2230) iscoco=3241.
if (isco08=2240) iscoco=3221.
if (isco08=2250) iscoco=2212.
if (isco08=2250) iscoco=2223.
if (isco08=2261) iscoco=2222.
if (isco08=2262) iscoco=2113.
if (isco08=2262) iscoco=2224.
if (isco08=2263) iscoco=2229.
if (isco08=2263) iscoco=2412.
if (isco08=2263) iscoco=3152.
if (isco08=2263) iscoco=3222.
if (isco08=2264) iscoco=3226.
if (isco08=2265) iscoco=3223.
if (isco08=2266) iscoco=3229.
if (isco08=2267) iscoco=3224.
if (isco08=2267) iscoco=3229.
if (isco08=2269) iscoco=2229.
if (isco08=2269) iscoco=3226.
if (isco08=2269) iscoco=3229.
if (isco08=2310) iscoco=2310.
if (isco08=2320) iscoco=2310.
if (isco08=2320) iscoco=2320.
if (isco08=2330) iscoco=2320.
if (isco08=2341) iscoco=2331.
if (isco08=2341) iscoco=3310.
if (isco08=2342) iscoco=2332.
if (isco08=2342) iscoco=3320.
if (isco08=2351) iscoco=2351.
if (isco08=2351) iscoco=2352.
if (isco08=2352) iscoco=2340.
if (isco08=2352) iscoco=3330.
if (isco08=2353) iscoco=2359.
if (isco08=2353) iscoco=3340.
if (isco08=2354) iscoco=2359.
if (isco08=2355) iscoco=2359.
if (isco08=2355) iscoco=3340.
if (isco08=2356) iscoco=2359.
if (isco08=2356) iscoco=3340.
```

```
if (isco08=2359) iscoco=2359.
if (isco08=2359) iscoco=3340.
if (isco08=2411) iscoco=2411.
if (isco08=2412) iscoco=2411.
if (isco08=2412) iscoco=2419.
if (isco08=2412) iscoco=3411.
if (isco08=2413) iscoco=2419.
if (isco08=2421) iscoco=2419.
if (isco08=2422) iscoco=2419.
if (isco08=2423) iscoco=2412.
if (isco08=2424) iscoco=2412.
if (isco08=2431) iscoco=2419.
if (isco08=2431) iscoco=2451.
if (isco08=2432) iscoco=2419.
if (isco08=2432) iscoco=2451.
if (isco08=2433) iscoco=3415.
if (isco08=2434) iscoco=3415.
if (isco08=2511) iscoco=2131.
if (isco08=2512) iscoco=2131.
if (isco08=2513) iscoco=2131.
if (isco08=2513) iscoco=2132.
if (isco08=2513) iscoco=2139.
if (isco08=2514) iscoco=2132.
if (isco08=2519) iscoco=2131.
if (isco08=2519) iscoco=2132.
if (isco08=2519) iscoco=2139.
if (isco08=2521) iscoco=2131.
if (isco08=2522) iscoco=2131.
if (isco08=2523) iscoco=2131.
if (isco08=2529) iscoco=2139.
if (isco08=2611) iscoco=2421.
if (isco08=2612) iscoco=2422.
if (isco08=2619) iscoco=2429.
if (isco08=2621) iscoco=2431.
if (isco08=2622) iscoco=2432.
if (isco08=2631) iscoco=2441.
if (isco08=2632) iscoco=2442.
if (isco08=2633) iscoco=2443.
if (isco08=2634) iscoco=2445.
if (isco08=2635) iscoco=2446.
if (isco08=2636) iscoco=2460.
if (isco08=2641) iscoco=2451.
if (isco08=2642) iscoco=2451.
if (isco08=2642) iscoco=3472.
if (isco08=2643) iscoco=2444.
if (isco08=2651) iscoco=2452.
if (isco08=2652) iscoco=2453.
if (isco08=2652) iscoco=3473.
if (isco08=2653) iscoco=2454.
if (isco08=2653) iscoco=3473.
if (isco08=2654) iscoco=1229.
```

```
if (isco08=2654) iscoco=2455.
if (isco08=2655) iscoco=2455.
if (isco08=2656) iscoco=3472.
if (isco08=2659) iscoco=3474.
if (isco08=3111) iscoco=3111.
if (isco08=3112) iscoco=3112.
if (isco08=3112) iscoco=3151.
if (isco08=3113) iscoco=3113.
if (isco08=3113) iscoco=3152.
if (isco08=3114) iscoco=3114.
if (isco08=3114) iscoco=3152.
if (isco08=3115) iscoco=3115.
if (isco08=3115) iscoco=3152.
if (isco08=3116) iscoco=3116.
if (isco08=3117) iscoco=3117.
if (isco08=3117) iscoco=3152.
if (isco08=3118) iscoco=3118.
if (isco08=3119) iscoco=3119.
if (isco08=3121) iscoco=7111.
if (isco08=3121) iscoco=8111.
if (isco08=3122) iscoco=8171.
if (isco08=3122) iscoco=8172.
if (isco08=3122) iscoco=8211.
if (isco08=3122) iscoco=8221.
if (isco08=3122) iscoco=8222.
if (isco08=3122) iscoco=8223.
if (isco08=3122) iscoco=8224.
if (isco08=3122) iscoco=8229.
if (isco08=3122) iscoco=8231.
if (isco08=3122) iscoco=8232.
if (isco08=3122) iscoco=8240.
if (isco08=3122) iscoco=8251.
if (isco08=3122) iscoco=8252.
if (isco08=3122) iscoco=8253.
if (isco08=3122) iscoco=8261.
if (isco08=3122) iscoco=8262.
if (isco08=3122) iscoco=8263.
if (isco08=3122) iscoco=8264.
if (isco08=3122) iscoco=8265.
if (isco08=3122) iscoco=8266.
if (isco08=3122) iscoco=8269.
if (isco08=3122) iscoco=8271.
if (isco08=3122) iscoco=8272.
if (isco08=3122) iscoco=8273.
if (isco08=3122) iscoco=8274.
if (isco08=3122) iscoco=8275.
if (isco08=3122) iscoco=8276.
if (isco08=3122) iscoco=8277.
if (isco08=3122) iscoco=8278.
if (isco08=3122) iscoco=8279.
if (isco08=3122) iscoco=8281.
```

```
if (isco08=3122) iscoco=8282.
if (isco08=3122) iscoco=8283.
if (isco08=3122) iscoco=8284.
if (isco08=3122) iscoco=8285.
if (isco08=3122) iscoco=8286.
if (isco08=3122) iscoco=8290.
if (isco08=3123) iscoco=1223.
if (isco08=3123) iscoco=7129.
if (isco08=3131) iscoco=8161.
if (isco08=3132) iscoco=8163.
if (isco08=3133) iscoco=8152.
if (isco08=3133) iscoco=8153.
if (isco08=3133) iscoco=8154.
if (isco08=3133) iscoco=8159.
if (isco08=3134) iscoco=8155.
if (isco08=3135) iscoco=8121.
if (isco08=3135) iscoco=8122.
if (isco08=3135) iscoco=8123.
if (isco08=3135) iscoco=8124.
if (isco08=3139) iscoco=3123.
if (isco08=3139) iscoco=8142.
if (isco08=3139) iscoco=8143.
if (isco08=3139) iscoco=8171.
if (isco08=3139) iscoco=8172.
if (isco08=3141) iscoco=3211.
if (isco08=3142) iscoco=3212.
if (isco08=3143) iscoco=3212.
if (isco08=3151) iscoco=3141.
if (isco08=3152) iscoco=3142.
if (isco08=3153) iscoco=3143.
if (isco08=3153) iscoco=3340.
if (isco08=3154) iscoco=3144.
if (isco08=3155) iscoco=3145.
if (isco08=3211) iscoco=3133.
if (isco08=3212) iscoco=3211.
if (isco08=3213) iscoco=3228.
if (isco08=3214) iscoco=7311.
if (isco08=3221) iscoco=2230.
if (isco08=3221) iscoco=3231.
if (isco08=3222) iscoco=2230.
if (isco08=3222) iscoco=3232.
if (isco08=3230) iscoco=3241.
if (isco08=3240) iscoco=3227.
if (isco08=3251) iscoco=3225.
if (isco08=3252) iscoco=4143.
if (isco08=3253) iscoco=3221.
if (isco08=3254) iscoco=3224.
if (isco08=3255) iscoco=3226.
if (isco08=3256) iscoco=3221.
if (isco08=3257) iscoco=3152.
if (isco08=3257) iscoco=3222.
```

```
if (isco08=3258) iscoco=5132.
if (isco08=3259) iscoco=3226.
if (isco08=3259) iscoco=3229.
if (isco08=3311) iscoco=3411.
if (isco08=3312) iscoco=3419.
if (isco08=3313) iscoco=3433.
if (isco08=3313) iscoco=3434.
if (isco08=3314) iscoco=3434.
if (isco08=3315) iscoco=3417.
if (isco08=3321) iscoco=3412.
if (isco08=3322) iscoco=3415.
if (isco08=3323) iscoco=3416.
if (isco08=3324) iscoco=3421.
if (isco08=3331) iscoco=3422.
if (isco08=3332) iscoco=3439.
if (isco08=3333) iscoco=3423.
if (isco08=3334) iscoco=3413.
if (isco08=3339) iscoco=2419.
if (isco08=3339) iscoco=3417.
if (isco08=3339) iscoco=3429.
if (isco08=3341) iscoco=3431.
if (isco08=3341) iscoco=4111.
if (isco08=3341) iscoco=4112.
if (isco08=3341) iscoco=4114.
if (isco08=3341) iscoco=4115.
if (isco08=3341) iscoco=4121.
if (isco08=3341) iscoco=4122.
if (isco08=3341) iscoco=4131.
if (isco08=3341) iscoco=4132.
if (isco08=3341) iscoco=4133.
if (isco08=3341) iscoco=4141.
if (isco08=3341) iscoco=4142.
if (isco08=3341) iscoco=4143.
if (isco08=3341) iscoco=4144.
if (isco08=3341) iscoco=4190.
if (isco08=3341) iscoco=4222.
if (isco08=3341) iscoco=4223.
if (isco08=3342) iscoco=3431.
if (isco08=3342) iscoco=4115.
if (isco08=3343) iscoco=3431.
if (isco08=3343) iscoco=3439.
if (isco08=3344) iscoco=3431.
if (isco08=3344) iscoco=4115.
if (isco08=3351) iscoco=3441.
if (isco08=3352) iscoco=3442.
if (isco08=3353) iscoco=3443.
if (isco08=3354) iscoco=3444.
if (isco08=3355) iscoco=3450.
if (isco08=3359) iscoco=3151.
if (isco08=3359) iscoco=3439.
if (isco08=3359) iscoco=3449.
```

```
if (isco08=3411) iscoco=3432.
if (isco08=3411) iscoco=3450.
if (isco08=3412) iscoco=3460.
if (isco08=3413) iscoco=3242.
if (isco08=3413) iscoco=3480.
if (isco08=3421) iscoco=3475.
if (isco08=3422) iscoco=3475.
if (isco08=3423) iscoco=3340.
if (isco08=3423) iscoco=3475.
if (isco08=3431) iscoco=3131.
if (isco08=3432) iscoco=3471.
if (isco08=3433) iscoco=3439.
if (isco08=3433) iscoco=3471.
if (isco08=3434) iscoco=5122.
if (isco08=3435) iscoco=1229.
if (isco08=3435) iscoco=3340.
if (isco08=3435) iscoco=3471.
if (isco08=3511) iscoco=3122.
if (isco08=3512) iscoco=3121.
if (isco08=3513) iscoco=3121.
if (isco08=3514) iscoco=3121.
if (isco08=3514) iscoco=3122.
if (isco08=3521) iscoco=3131.
if (isco08=3521) iscoco=3132.
if (isco08=3522) iscoco=3114.
if (isco08=3522) iscoco=3132.
if (isco08=4110) iscoco=4190.
if (isco08=4120) iscoco=4115.
if (isco08=4131) iscoco=4111.
if (isco08=4131) iscoco=4112.
if (isco08=4132) iscoco=4113.
if (isco08=4132) iscoco=4114.
if (isco08=4211) iscoco=4211.
if (isco08=4211) iscoco=4212.
if (isco08=4212) iscoco=4211.
if (isco08=4212) iscoco=4213.
if (isco08=4213) iscoco=4214.
if (isco08=4214) iscoco=4215.
if (isco08=4221) iscoco=3414.
if (isco08=4221) iscoco=4221.
if (isco08=4222) iscoco=4222.
if (isco08=4223) iscoco=4223.
if (isco08=4224) iscoco=4222.
if (isco08=4225) iscoco=4222.
if (isco08=4226) iscoco=4222.
if (isco08=4227) iscoco=4190.
if (isco08=4229) iscoco=4222.
if (isco08=4311) iscoco=4121.
if (isco08=4312) iscoco=4122.
if (isco08=4313) iscoco=4121.
if (isco08=4321) iscoco=4131.
```

```
if (isco08=4322) iscoco=4132.
if (isco08=4323) iscoco=4133.
if (isco08=4411) iscoco=4141.
if (isco08=4412) iscoco=4142.
if (isco08=4413) iscoco=4143.
if (isco08=4414) iscoco=4144.
if (isco08=4415) iscoco=4141.
if (isco08=4416) iscoco=4190.
if (isco08=4419) iscoco=4190.
if (isco08=5111) iscoco=5111.
if (isco08=5112) iscoco=5112.
if (isco08=5113) iscoco=5113.
if (isco08=5120) iscoco=5122.
if (isco08=5131) iscoco=5123.
if (isco08=5132) iscoco=5123.
if (isco08=5141) iscoco=5141.
if (isco08=5142) iscoco=5141.
if (isco08=5151) iscoco=5121.
if (isco08=5152) iscoco=5121.
if (isco08=5153) iscoco=9141.
if (isco08=5161) iscoco=5151.
if (isco08=5161) iscoco=5152.
if (isco08=5162) iscoco=5142.
if (isco08=5163) iscoco=5143.
if (isco08=5164) iscoco=5139.
if (isco08=5164) iscoco=6129.
if (isco08=5165) iscoco=3340.
if (isco08=5169) iscoco=5149.
if (isco08=5211) iscoco=5230.
if (isco08=5212) iscoco=9111.
if (isco08=5221) iscoco=1314.
if (isco08=5222) iscoco=5220.
if (isco08=5223) iscoco=5220.
if (isco08=5230) iscoco=4211.
if (isco08=5241) iscoco=5210.
if (isco08=5242) iscoco=5220.
if (isco08=5243) iscoco=9113.
if (isco08=5244) iscoco=9113.
if (isco08=5245) iscoco=5220.
if (isco08=5246) iscoco=5220.
if (isco08=5246) iscoco=5230.
if (isco08=5249) iscoco=5220.
if (isco08=5311) iscoco=5131.
if (isco08=5312) iscoco=5131.
if (isco08=5321) iscoco=5132.
if (isco08=5322) iscoco=5133.
if (isco08=5329) iscoco=5132.
if (isco08=5329) iscoco=5139.
if (isco08=5411) iscoco=5161.
if (isco08=5412) iscoco=5162.
if (isco08=5413) iscoco=5163.
```

```
if (isco08=5414) iscoco=5169.
if (isco08=5414) iscoco=9152.
if (isco08=5419) iscoco=5169.
if (isco08=6111) iscoco=1311.
if (isco08=6111) iscoco=6111.
if (isco08=6112) iscoco=1311.
if (isco08=6112) iscoco=6112.
if (isco08=6113) iscoco=1311.
if (isco08=6113) iscoco=6113.
if (isco08=6114) iscoco=1311.
if (isco08=6114) iscoco=6114.
if (isco08=6121) iscoco=1311.
if (isco08=6121) iscoco=6121.
if (isco08=6121) iscoco=6124.
if (isco08=6122) iscoco=1311.
if (isco08=6122) iscoco=6122.
if (isco08=6122) iscoco=6124.
if (isco08=6123) iscoco=6123.
if (isco08=6123) iscoco=6124.
if (isco08=6129) iscoco=6129.
if (isco08=6130) iscoco=1311.
if (isco08=6130) iscoco=6130.
if (isco08=6210) iscoco=1311.
if (isco08=6210) iscoco=6141.
if (isco08=6210) iscoco=6142.
if (isco08=6221) iscoco=1311.
if (isco08=6221) iscoco=6151.
if (isco08=6222) iscoco=1311.
if (isco08=6222) iscoco=6152.
if (isco08=6223) iscoco=1311.
if (isco08=6223) iscoco=6153.
if (isco08=6224) iscoco=6154.
if (isco08=6310) iscoco=6210.
if (isco08=6320) iscoco=6210.
if (isco08=6330) iscoco=6210.
if (isco08=6340) iscoco=6210.
if (isco08=7111) iscoco=7121.
if (isco08=7111) iscoco=7129.
if (isco08=7112) iscoco=7122.
if (isco08=7113) iscoco=7113.
if (isco08=7113) iscoco=7122.
if (isco08=7114) iscoco=7123.
if (isco08=7115) iscoco=7124.
if (isco08=7119) iscoco=7129.
if (isco08=7121) iscoco=7131.
if (isco08=7122) iscoco=7132.
if (isco08=7123) iscoco=7133.
if (isco08=7124) iscoco=7134.
if (isco08=7125) iscoco=7135.
if (isco08=7126) iscoco=7136.
if (isco08=7127) iscoco=7233.
```

```
if (isco08=7131) iscoco=7141.
if (isco08=7132) iscoco=7142.
if (isco08=7133) iscoco=7143.
if (isco08=7211) iscoco=7211.
if (isco08=7212) iscoco=7212.
if (isco08=7213) iscoco=7213.
if (isco08=7214) iscoco=7214.
if (isco08=7215) iscoco=7215.
if (isco08=7221) iscoco=7221.
if (isco08=7222) iscoco=7222.
if (isco08=7223) iscoco=7223.
if (isco08=7223) iscoco=8211.
if (isco08=7224) iscoco=7224.
if (isco08=7231) iscoco=7231.
if (isco08=7232) iscoco=7232.
if (isco08=7233) iscoco=7233.
if (isco08=7234) iscoco=7231.
if (isco08=7311) iscoco=7311.
if (isco08=7312) iscoco=7312.
if (isco08=7313) iscoco=7313.
if (isco08=7314) iscoco=7321.
if (isco08=7315) iscoco=7322.
if (isco08=7316) iscoco=7323.
if (isco08=7316) iscoco=7324.
if (isco08=7317) iscoco=7331.
if (isco08=7317) iscoco=7424.
if (isco08=7318) iscoco=7332.
if (isco08=7318) iscoco=7431.
if (isco08=7318) iscoco=7432.
if (isco08=7319) iscoco=7331.
if (isco08=7321) iscoco=7341.
if (isco08=7321) iscoco=7342.
if (isco08=7321) iscoco=7343.
if (isco08=7322) iscoco=7341.
if (isco08=7322) iscoco=7346.
if (isco08=7322) iscoco=8251.
if (isco08=7323) iscoco=7345.
if (isco08=7323) iscoco=8252.
if (isco08=7411) iscoco=7137.
if (isco08=7412) iscoco=7241.
if (isco08=7413) iscoco=7245.
if (isco08=7421) iscoco=7242.
if (isco08=7421) iscoco=7243.
if (isco08=7422) iscoco=7242.
if (isco08=7422) iscoco=7243.
if (isco08=7422) iscoco=7244.
if (isco08=7422) iscoco=7245.
if (isco08=7511) iscoco=7411.
if (isco08=7512) iscoco=7412.
if (isco08=7513) iscoco=7413.
if (isco08=7514) iscoco=7414.
```

```
if (isco08=7515) iscoco=7415.
if (isco08=7516) iscoco=7416.
if (isco08=7521) iscoco=7421.
if (isco08=7522) iscoco=7422.
if (isco08=7523) iscoco=7423.
if (isco08=7523) iscoco=8240.
if (isco08=7531) iscoco=7433.
if (isco08=7531) iscoco=7434.
if (isco08=7532) iscoco=7435.
if (isco08=7533) iscoco=7436.
if (isco08=7534) iscoco=7437.
if (isco08=7535) iscoco=7441.
if (isco08=7536) iscoco=7442.
if (isco08=7541) iscoco=6152.
if (isco08=7541) iscoco=7216.
if (isco08=7542) iscoco=7112.
if (isco08=7543) iscoco=3152.
if (isco08=7544) iscoco=7143.
if (isco08=7549) iscoco=7322.
if (isco08=8111) iscoco=7111.
if (isco08=8111) iscoco=8111.
if (isco08=8112) iscoco=8112.
if (isco08=8113) iscoco=8113.
if (isco08=8114) iscoco=8212.
if (isco08=8121) iscoco=8121.
if (isco08=8121) iscoco=8122.
if (isco08=8121) iscoco=8123.
if (isco08=8121) iscoco=8124.
if (isco08=8122) iscoco=8223.
if (isco08=8131) iscoco=8151.
if (isco08=8131) iscoco=8152.
if (isco08=8131) iscoco=8153.
if (isco08=8131) iscoco=8154.
if (isco08=8131) iscoco=8155.
if (isco08=8131) iscoco=8159.
if (isco08=8131) iscoco=8221.
if (isco08=8131) iscoco=8222.
if (isco08=8131) iscoco=8229.
if (isco08=8132) iscoco=7344.
if (isco08=8132) iscoco=8224.
if (isco08=8141) iscoco=8231.
if (isco08=8142) iscoco=8232.
if (isco08=8143) iscoco=8253.
if (isco08=8151) iscoco=8261.
if (isco08=8152) iscoco=7432.
if (isco08=8152) iscoco=8262.
if (isco08=8153) iscoco=8263.
if (isco08=8154) iscoco=8264.
if (isco08=8155) iscoco=8265.
if (isco08=8156) iscoco=8266.
if (isco08=8157) iscoco=8264.
```

```
if (isco08=8159) iscoco=8269.
if (isco08=8160) iscoco=8271.
if (isco08=8160) iscoco=8272.
if (isco08=8160) iscoco=8273.
if (isco08=8160) iscoco=8274.
if (isco08=8160) iscoco=8275.
if (isco08=8160) iscoco=8276.
if (isco08=8160) iscoco=8277.
if (isco08=8160) iscoco=8278.
if (isco08=8160) iscoco=8279.
if (isco08=8171) iscoco=8142.
if (isco08=8171) iscoco=8143.
if (isco08=8172) iscoco=8141.
if (isco08=8181) iscoco=8131.
if (isco08=8181) iscoco=8139.
if (isco08=8182) iscoco=8162.
if (isco08=8183) iscoco=8290.
if (isco08=8189) iscoco=8290.
if (isco08=8211) iscoco=8281.
if (isco08=8212) iscoco=8282.
if (isco08=8212) iscoco=8283.
if (isco08=8219) iscoco=8284.
if (isco08=8219) iscoco=8285.
if (isco08=8219) iscoco=8286.
if (isco08=8219) iscoco=8290.
if (isco08=8311) iscoco=8311.
if (isco08=8312) iscoco=8312.
if (isco08=8321) iscoco=8321.
if (isco08=8322) iscoco=8322.
if (isco08=8331) iscoco=8323.
if (isco08=8332) iscoco=8324.
if (isco08=8341) iscoco=8331.
if (isco08=8342) iscoco=8332.
if (isco08=8343) iscoco=8333.
if (isco08=8344) iscoco=8334.
if (isco08=8350) iscoco=8340.
if (isco08=9111) iscoco=9131.
if (isco08=9112) iscoco=9132.
if (isco08=9121) iscoco=9133.
if (isco08=9122) iscoco=9142.
if (isco08=9123) iscoco=9142.
if (isco08=9129) iscoco=9142.
if (isco08=9211) iscoco=9211.
if (isco08=9212) iscoco=9211.
if (isco08=9213) iscoco=9211.
if (isco08=9214) iscoco=6113.
if (isco08=9214) iscoco=9211.
if (isco08=9215) iscoco=9212.
if (isco08=9216) iscoco=9213.
if (isco08=9311) iscoco=9311.
if (isco08=9312) iscoco=9312.
```

```
if (isco08=9313) iscoco=9313.
if (isco08=9321) iscoco=9322.
if (isco08=9329) iscoco=9321.
if (isco08=9329) iscoco=9322.
if (isco08=9331) iscoco=9331.
if (isco08=9332) iscoco=9332.
if (isco08=9333) iscoco=9333.
if (isco08=9334) iscoco=9333.
if (isco08=9411) iscoco=5122.
if (isco08=9412) iscoco=9132.
if (isco08=9510) iscoco=9120.
if (isco08=9520) iscoco=9112.
if (isco08=9611) iscoco=9161.
if (isco08=9612) iscoco=9161.
if (isco08=9612) iscoco=9321.
if (isco08=9613) iscoco=9162.
if (isco08=9621) iscoco=9151.
if (isco08=9621) iscoco=9152.
if (isco08=9622) iscoco=9162.
if (isco08=9623) iscoco=9153.
if (isco08=9624) iscoco=9162.
if (isco08=9629) iscoco=9152.
if (isco08=0110) iscoco=0110.
if (isco08=0210) iscoco=0110.
if (isco08=0310) iscoco=0110.
IF  (isco08 ~= 66666 AND isco08 ~= 77777 AND isco08 ~= 88888 AND isco08 ~= 99999 AND
SYSMIS(iscoco))
   iscoco=isco08.
EXECUTE.

*Assigning EGP values to each of the ISCO codes

RECODE iscoco (100=1) (110=1) (111=1) (112=1) (122=1) (1000 thru 1120=1) (1200=1) (1210=1)
(1220=1) (1222 thru 1239=1) (1250=1) (1251=1) (2000 thru 2131=1) (2140 thru 2147=1) (2149 thru
2229=1) (2310=1) (2311=1)
(235=1) (2350=1) (2351=1) (2352=1) (2400=1) (2411=1) (2420 thru 2429=1) (2440 thru 2443=1)
(2445=1) (3143=1) (3144=1)
(1240=2) (1130 thru 1143=2) (1252=2) (1300=2) (1310=2) (131=2) (1312 thru 1320=2) (2132=2)
(2139=2) (2148=2) (2230 thru 2236=2) (2300=2) (2320 thru 2340=2)
(2359=2) (2410=2) (2412=2) (2413=2) (2414=2) (2419=2) (2430=2) (2431=2) (2432=2) (2444=2) (2446
thru 3142=2) (3145 thru 3229=2) (322=2) (5135=2) (3240=2)
(3241=2) (3242=2) (3400 thru 3429=2) (3431=2) (3432=2) (3434=2) (3440 thru 3451=2) (3470 thru
3475=2) (5121=2) (5150=2) (5151=2) (5152=2) (3230 thru 3239=3) (3300 thru 3340=3)
(3430=3) (3433=3) (3439=3) (3452=3) (3460=3) (3461=3) (3462=3) (411 412=3) (3480 thru 4141=3)
(422=3) (4143 thru 5120=3) (5131=3) (5133=3) (5134=3) (5200 thru 5230=3) (7510=3) (9100 thru
9113=3)
(5122=4) (5140=4) (5141=4) (5143=4) (5161=4) (5162=4) (5164=4) (7000=4) (7120=4) (7124=4)
(7129=4) (7130=4) (7132=4) (7133=4) (7134=4) (7136 thru 7141=4) (7200 thru 7233=4)
(7240 thru 7313=4) (7323=4) (7324=4) (7340 thru 7420=4) (7422=4) (7423=4) (7430=4) (7433 thru
7500=4) (7520=4) (816=4) (8150 thru 8172=4) (8311=4) (8332=4) (8333=4)
```

```
(513=5) (5123=5) (5130=5) (4142=5) (5132=5) (5139=5) (5142=5) (5149=5) (516=5) (5160=5)
(5153=5) (5163=5) (5169=5) (6000 thru 6130=5) (614=5)
(6134 thru 6154=5) (7100 thru 7113=5) (7121=5) (7122=5) (7123=5) (7131=5) (7135=5) (7142=5)
(7143=5) (7234=5) (7320=5) (7321=5) (7322=5) (733=5) (7330=5) (7331=5)
(7332=5) (7421=5) (7424=5) (7431=5) (7432=5) (7530 thru 8144=5) (8200 thru 8310=5) (8312 thru
8331=5) (8334 thru 9000=5) (916=5) (931=5) (9120 thru 9333=5)
(1221=6) (1311=6) (6131=6) (6132=6) (6133=6) (6200=6) (6210=6) into egpclass.
EXECUTE.

*Constructing the EGP classes on the basis of the main activity last 7 days

IF (emplrel=2 OR emplrel=3 AND egpclass >= 2) egpclass=6.
Execute.

if (pdwrk=1) activ = 1.
if (edctn=1) activ = 2.
if (uempla=1) activ = 3.
IF (uempli=1) activ = 4.
if (dsbld=1) activ = 5.
if (rtrd=1) activ = 6.
if (cmsrv=1) activ = 7.
if (hswrk=1) activ = 8.
IF (dngoth=1) activ = 9.
IF (mainact >= 1 AND mainact<=9) activ=mainact.
EXECUTE.

IF (egpclass = 1 AND activ=1) EGP=1.
IF (egpclass = 2 OR egpclass = 3 AND activ=1) EGP= 2.
IF (egpclass = 4 OR egpclass = 5 AND activ=1) EGP = 3.
If (egpclass = 6 AND activ=1) EGP = 4.
IF (activ = 3 OR activ= 4) EGP = 5.
IF (activ = 2 OR  activ = 5 OR  activ = 6 OR  activ = 7 OR  activ = 8 OR activ = 9) EGP = 6.
EXECUTE.

*Creating dummies for each of the EGP classes

RECODE EGP (1=1) (MISSING=SYSMIS) (ELSE=0) INTO Dienst.
RECODE EGP (2=1) (MISSING=SYSMIS) (ELSE=0) INTO Hoofd.
RECODE EGP (3=1) (MISSING=SYSMIS) (ELSE=0) INTO Hand.
RECODE EGP (4=1) (MISSING=SYSMIS) (ELSE=0) INTO Zelf.
RECODE EGP (5=1) (MISSING=SYSMIS) (ELSE=0) INTO Werkloos.
RECODE EGP (6=1) (MISSING=SYSMIS) (ELSE=0) INTO Pensioen.
EXECUTE.

*Dividing education level in three groups (two dummies)

RECODE edulvlb (MISSING=SYSMIS) (0 thru 299=1) (300 thru 499=2) (500 thru Highest=3) INTO educ.
EXECUTE.

RECODE educ (MISSING=SYSMIS) (1=1) (ELSE=0) INTO Lager.
RECODE educ (MISSING=SYSMIS) (3=1) (ELSE=0) INTO Hoger.
```

```
EXECUTE.

*Creating a dummy for whether someone lives in a urbanized or rural region

RECODE domicil (MISSING=SYSMIS) (1 thru 3=1) (ELSE=0) INTO city.
EXECUTE.

*Recoding gender to a dummy variable

RECODE gndr (MISSING=SYSMIS) (1=1) (ELSE=0) INTO gender.
EXECUTE.

*Recoding feelings of unsafety into a dummy variable

RECODE aesfdrk (MISSING=SYSMIS) (1 thru 2=1) (ELSE=0) INTO Safe.
EXECUTE.

*Reversing the items of the human values so that higher values point to higher identification with
the relevant human value

RECODE ipfrule ipbhprp imptrad ipmodst ipeqopt ipudrst impenv impsafe ipstrgv (MISSING=SYSMIS)
(1=6) (2=5) (3=4) (4=3)
    (5=2) (6=1) INTO sipfrule sipbhprp simptrad sipmodst sipeqopt sipudrst simpenv simpsafe sipstrgv.
EXECUTE.

*Reversing the economic threat and cultural threat item so that higher values point to higher levels
of ethnic threat

COMPUTE simbgeco=10 - imbgeco.
COMPUTE simueclt=10- imueclt.
EXECUTE.

*Constructing three dummy variables for subjective income

RECODE hincfel (2=1) (MISSING=SYSMIS) (ELSE=0) INTO coping.
RECODE hincfel (3=1) (MISSING=SYSMIS) (ELSE=0) INTO diff.
RECODE hincfel (4=1) (MISSING=SYSMIS) (ELSE=0) INTO very.
EXECUTE.

*Coding additional indicators, which are not used in the final model

RECODE rfgfrpc (MISSING=SYSMIS) (1=5) (2=4) (3=3) (4=2) (5=1) INTO srfgfrpc.
EXECUTE.

IF  (diff = 1 OR very=1) difver=1.
IF  (diff  ~= 1 AND very  ~=1) difver=0.
IF  (MISSING(diff) = 1) difver=$SYSMIS.
EXECUTE.

COMPUTE simwbcnt=10- imwbcnt.
EXECUTE.
```

*CONSTUCTING CONTEXTUAL VARIABLES

*Constructing country codes

```
RECODE cntry ('AT'=1) ('BE'=2) ('CH'=3) ('CZ'=4) ('DE'=5) ('EE'=6) ('ES'=7) ('FI'=8) ('FR'=9)
('GB'=10) ('IE'=11) ('IL'=12) ('IS'=13) ('IT'=14) ('LT'=15) ('NL'=16) ('NO'=17) ('PL'=18) ('PT'=19)
('RU'=20) ('SE'=21) ('SI'=22) INTO country.
EXECUTE.
```

*Assigning values for long-term unemployment rate 2011-2016

```
IF  (country=1) unemploy1116=5.35.
IF  (country=2) unemploy1116=7.99.
IF  (country=3) unemploy1116=4.7.
IF  (country=4) unemploy1116=5.96.
IF  (country=5) unemploy1116=5.03.
IF  (country=6) unemploy1116=8.55.
IF  (country=7) unemploy1116=23.07.
IF  (country=8) unemploy1116=8.42.
IF  (country=9) unemploy1116=9.81.
IF  (country=10) unemploy1116=6.61.
IF  (country=11) unemploy1116=11.81.
IF  (country=13) unemploy1116=5.05.
IF  (country=14) unemploy1116=11.24.
IF  (country=15) unemploy1116=11.37.
IF  (country=16) unemploy1116=6.39.
IF  (country=17) unemploy1116=3.7.
IF  (country=18) unemploy1116=8.78.
IF  (country=19) unemploy1116=13.63.
IF  (country=21) unemploy1116=7.68.
IF  (country=22) unemploy1116=8.96.
EXECUTE.
```

*Assigning values for recognition rate

```
IF  (country=1) accept=71.602.
IF  (country=2) accept=60.176.
IF  (country=3) accept=58.350.
IF  (country=4) accept=33.333.
IF  (country=5) accept=68.746.
IF  (country=6) accept=68.421.
IF  (country=7) accept=66.845.
IF  (country=8) accept=34.048.
IF  (country=9) accept=32.869.
IF  (country=10) accept=32.028.
IF  (country=11) accept=22.770.
IF  (country=13) accept=17.593.
IF  (country=14) accept=39.394.
IF  (country=15) accept=69.643.
IF  (country=16) accept=72.069.
```

```
IF  (country=17) accept=66.064.
IF  (country=18) accept=12.224.
IF  (country=19) accept=53.782.
IF  (country=21) accept=69.477.
IF  (country=22) accept=64.151.
EXECUTE.

*Assigning values for rate of asylum applicants from Middle Eastern conflict regions

IF  (country=1) rate=55.410.
IF  (country=2) rate=34.570.
IF  (country=3) rate=24.563.
IF  (country=4) rate=18.243.
IF  (country=5) rate=66.252.
IF  (country=6) rate=42.857.
IF  (country=7) rate=20.184.
IF  (country=8) rate=45.956.
IF  (country=9) rate=15.940.
IF  (country=10) rate=21.403.
IF  (country=11) rate=20.713.
IF  (country=13) rate=12.000.
IF  (country=14) rate=4.355.
IF  (country=15) rate=54.651.
IF  (country=16) rate=25.830.
IF  (country=17) rate=33.097.
IF  (country=18) rate=0.853.
IF  (country=19) rate=38.225.
IF  (country=21) rate=38.687.
IF  (country=22) rate=62.595.
EXECUTE.

*Assigning values for number of asylum applicants per 1000 inhabitants

IF  (country=1) asyltot=4.866.
IF  (country=2) asyltot=1.620.
IF  (country=3) asyltot=3.266.
IF  (country=4) asyltot=0.140.
IF  (country=5) asyltot=9.069.
IF  (country=6) asyltot=0.133.
IF  (country=7) asyltot=0.339.
IF  (country=8) asyltot=1.025.
IF  (country=9) asyltot=1.262.
IF  (country=10) asyltot=0.610.
IF  (country=11) asyltot=0.475.
IF  (country=13) asyltot=3.383.
IF  (country=14) asyltot=2.027.
IF  (country=15) asyltot=0.149.
IF  (country=16) asyltot=1.234.
IF  (country=17) asyltot=0.676.
IF  (country=18) asyltot=0.324.
IF  (country=19) asyltot=0.142.
```

```
IF  (country=21) asyltot=2.930.
IF  (country=22) asyltot=0.635.
EXECUTE.

*Assigning values for number of asylum applicants per 1000 inhabitants 2015 and 2016 combined

IF  (country=1) asyl1516=7.574.
IF  (country=2) asyl1516=2.802.
IF  (country=3) asyl1516=4.031.
IF  (country=4) asyl1516=0.142.
IF  (country=5) asyl1516=7.470.
IF  (country=6) asyl1516=0.154.
IF  (country=7) asyl1516=0.329.
IF  (country=8) asyl1516=3.468.
IF  (country=9) asyl1516=1.204.
IF  (country=10) asyl1516=0.616.
IF  (country=11) asyl1516=0.591.
IF  (country=13) asyl1516=2.254.
IF  (country=14) asyl1516=1.700.
IF  (country=15) asyl1516=0.128.
IF  (country=16) asyl1516=1.947.
IF  (country=17) asyl1516=3.352.
IF  (country=18) asyl1516=0.322.
IF  (country=19) asyl1516=0.114.
IF  (country=21) asyl1516=9.803.
IF  (country=22) asyl1516=0.384.
EXECUTE.

*Assigning values for change in unemployment rate between 2006 and 2016

IF  (country=1) dunemploy0616=0.8.
IF  (country=2) dunemploy0616=-0.4.
IF  (country=3) dunemploy0616=0.9.
IF  (country=4) dunemploy0616=-3.1.
IF  (country=5) dunemploy0616=-6.2.
IF  (country=6) dunemploy0616=0.9.
IF  (country=7) dunemploy0616=11.1.
IF  (country=8) dunemploy0616=1.1.
IF  (country=9) dunemploy0616=1.7.
IF  (country=10) dunemploy0616=-0.6.
IF  (country=11) dunemploy0616=4.
IF  (country=13) dunemploy0616=0.2.
IF  (country=14) dunemploy0616=4.9.
IF  (country=15) dunemploy0616=2.1.
IF  (country=16) dunemploy0616=2.1.
IF  (country=17) dunemploy0616=1.3.
IF  (country=18) dunemploy0616=-7.6.
IF  (country=19) dunemploy0616=3.5.
IF  (country=21) dunemploy0616=-0.1.
IF  (country=22) dunemploy0616=2.
EXECUTE.
```

*Assigning values for percentage of foreign born individuals from outside the EU

```
IF  (country=1) foreign16=10.059.
IF  (country=2) foreign16=8.654.
IF  (country=3) foreign16=11.179.
IF  (country=4) foreign16=2.477.
IF  (country=5) foreign16=7.979.
IF  (country=6) foreign16=13.271.
IF  (country=7) foreign16=8.531.
IF  (country=8) foreign16=3.834.
IF  (country=9) foreign16=8.664.
IF  (country=10) foreign16=8.331.
IF  (country=11) foreign16=3.855.
IF  (country=13) foreign16=4.124.
IF  (country=14) foreign16=6.731.
IF  (country=15) foreign16=3.769.
IF  (country=16) foreign16=8.844.
IF  (country=17) foreign16=8.143.
IF  (country=18) foreign16=1.080.
IF  (country=19) foreign16=6.193.
IF  (country=21) foreign16=11.625.
IF  (country=22) foreign16=8.439.
EXECUTE.
```

*Saving the dataset with only the relevant variables

```
SAVE OUTFILE='C:\Users\u0110527\Desktop\ISPO\PUMOMIG\Multilevel paper\Data\Multilevel
reviewed.sav'
  /keep idno cntry
 lrscale stfeco imsmetn imdfetn
 impcntr imbgeco imueclt imwbcnt rlgdgr gvrfgap rfgbfml
 uemplwk hinctnta hincfel dweight Lager Hoger
 Dienst Hoofd Hand Zelf Werkloos
 Pensioen city gender safe srfgfrpc sipfrule sipbhprp simptrad
 sipmodst sipeqopt sipudrst simpenv simpsafe sipstrgv simbgeco
 simueclt simwbcnt coping diff very difver agea country
 unemploy1116 accept rate asyltot asyl1516 dunemploy0616 foreign16
  /COMPRESSED.
```

*Recoding missings values into the code -99

```
RECODE lrscale stfeco imsmetn imdfetn impcntr imbgeco imueclt imwbcnt rlgdgr gvrfgap rfgbfml
   uemplwk hinctnta hincfel dweight Lager Hoger Dienst Hoofd Hand Zelf Werkloos Pensioen city
gender
   Safe srfgfrpc sipfrule sipbhprp simptrad sipmodst sipeqopt sipudrst simpenv simpsafe sipstrgv
   simbgeco simueclt simwbcnt coping diff very difver agea country unemploy1116 accept rate
asyltot
   asyl1516 dunemploy0616 foreign16 (MISSING=-99).
EXECUTE.
```

```
*Saving dat.file for mplus analysis

SAVE TRANSLATE OUTFILE='C:\Users\u0110527\Desktop\ISPO\PUMOMIG\Multilevel
paper\analyses\multilevel_reviewed.dat'
  /TYPE=TAB
  /ENCODING='UTF8'
  /MAP
  /REPLACE
  /CELLS=VALUES.
```

Mplus syntax

```
Title:
  Model

Data:
  File is "C:\Users\u0110527\Desktop\Multilevel paper\analyses\multilevel_reviewed.dat";

Variable:

 NAMES ARE idno cntry
 lrscale stfeco imsmetn imdfetn
 impcntr imbgeco imueclt imwbcnt rlgdgr gvrfgap rfgbfml
 uemplwk hinctnta hincfel dweight Lager Hoger
 Dienst Hoofd Hand Zelf Werkloos
 Pensioen city gender safe srfgfrpc sipfrule sipbhprp simptrad
 sipmodst sipeqopt sipudrst simpenv simpsafe sipstrgv simbgeco
 simueclt simwbcnt coping diff very difver agea country
 unemploy1116 accept rate asyltot asyl1516 dunemploy0616 foreign16;

 USEVARIABLES ARE
 simbgeco simueclt gvrfgap rfgbfml
 rlgdgr agea
 Lager Hoger Dienst Hoofd Zelf Werkloos
 Pensioen  gender safe city
 sipfrule sipbhprp simptrad
 sipmodst sipeqopt sipudrst simpenv
 coping diff very asyltot unemploy1116 accept rate ;

 WITHIN is simbgeco simueclt
 rlgdgr agea
 Lager Hoger Dienst Hoofd Zelf Werkloos
 Pensioen gender safe city
 sipfrule sipbhprp simptrad
 sipmodst sipeqopt sipudrst simpenv
 coping diff very ;

 BETWEEN is accept unemploy1116 asyltot rate;

 CLUSTER=country ;

 USEOBSERVATIONS ARE (country EQ 1 OR country EQ 2 OR country EQ 3
 OR country EQ 4 OR country EQ 5 OR country EQ 6 OR country EQ 7
 OR country EQ 8
 OR country EQ 9 OR country EQ 10 OR country EQ 11
 OR country EQ 13 OR country EQ 14 OR country EQ 15 OR country EQ 16
 OR country EQ 17 OR country EQ 18 OR country EQ 19
 OR country EQ 21 OR country EQ 22) ;

 MISSING ARE all (-99) ;
```

```
 ANALYSIS:
TYPE = TWOLEVEL ;
!ESTIMATOR = MLR ;
 ESTIMATOR IS BAYES ;
 ALGORITHM=GIBBS;
 BCONVERGENCE = 0.01 ;
 thin=50 ;
fbiterations=10000 ;
process = 2;

 MODEL:

 %WITHIN%

ref by gvrfgap
rfgbfml (a);

Contrad by sipfrule sipbhprp simptrad sipmodst ;
Univer by sipeqopt sipudrst simpenv ;
Contrad WITH Univer ;

SIMPENV  WITH SIMPTRAD ;
SIPUDRST WITH SIPMODST ;

 simbgeco with simueclt ;

 ref on
 contrad univer
 simbgeco simueclt
 rlgdgr agea lager hoger  Gender
 Dienst hoofd
 zelf Werkloos Pensioen  coping diff very
 safe city ;

 Contrad on rlgdgr agea lager hoger  Gender
 Dienst hoofd
 zelf Werkloos Pensioen  coping diff very
 safe city ;

 Univer on rlgdgr agea lager hoger  Gender
 Dienst hoofd
 zelf Werkloos Pensioen  coping diff very
 safe city ;

 simbgeco on contrad univer
 rlgdgr agea lager hoger  Gender
 Dienst hoofd
 zelf Werkloos Pensioen  coping diff very
 safe city;
```

```
 simueclt on contrad univer
 rlgdgr agea lager hoger  Gender
 Dienst hoofd
 zelf Werkloos Pensioen coping diff very
 safe city;


 %BETWEEN%
refb by gvrfgap
rfgbfml (a) ;

 refb on accept unemploy1116 asyltot rate ;

 OUTPUT:

 cinterval tech8 standardized tech1 sampstat ;

 plot: type = plot2 ;
```
