# Supplementary material for: Attitudes Toward Asylum Policy in a Divided Europe: Diverging Contexts, Diverging Attitudes?
Source: Front Sociol. 2020 May 21;5:35. doi: 10.3389/fsoc.2020.00035 (PMC8022488; doi:10.3389/fsoc.2020.00035)
Supplement: Supplementary file 2 [file Table_1.docx]

| - Table A1. Sample sizes and contextual variables per country (ESS8) | | | | | | | | | |
| --- | --- | --- | --- | --- | --- | --- | --- | --- | --- |
| - **Country** | **Sample size** | **Mean attitudes asylum policy** | - **Unemployment 2011-2016** | - **Recognition rate** | **Asylum applications 2016** | **Rate from Syria, Iraq or Afghanistan** | **Asylum applications 2015-2016** | **Foreign-born population** | **Change unemployment 2006-2016** |
| - Austria | 1746 | 3.24 | 5.35% | 71.60% | 4.87 | 55.41% | 7.57 | 10.06% | 0.80 |
| - Belgium | 1467 | 3.26 | 7.99% | 60.18% | 1.62 | 34.57% | 2.80 | 8.65% | -0.40 |
| - Czech Republic | 2176 | 3.50 | 5.96% | 33.33% | 0.14 | 18.24% | 0.14 | 2.48% | -3.10 |
| - Estonia | 1524 | 3.50 | 8.55% | 68.42% | 0.13 | 42.86% | 0.15 | 13.27% | 0.90 |
| - Finland | 1820 | 2.59 | 8.42% | 34.05% | 1.03 | 45.96% | 3.47 | 3.83% | 1.10 |
| - France | 1790 | 2.71 | 9.81% | 32.87% | 1.26 | 15.94% | 1.20 | 8.66% | 1.70 |
| - Germany | 2477 | 3.00 | 5.03% | 68.75% | 9.07 | 66.25% | 7.47 | 7.98% | -6.20 |
| - Iceland | 807 | 2.35 | 5.05% | 17.59% | 3.83 | 12.00% | 2.25 | 4.12% | 0.20 |
| - Ireland | 2266 | 2.66 | 11.81% | 22.77% | 0.48 | 20.71% | 0.59 | 3.86% | 4.00 |
| - Italy | 2341 | 3.04 | 11.24% | 39.39% | 2.03 | 4.36% | 1.70 | 6.73% | 4.90 |
| - Lithuania | 1884 | 2.84 | 11.37% | 69.64% | 0.15 | 54.65% | 0.13 | 3.77% | 2.10 |
| - Netherlands | 1512 | 3.36 | 6.39% | 72.07% | 1.23 | 25.83% | 1.95 | 8.84% | 2.10 |
| - Norway | 1334 | 2.60 | 3.70% | 66.06% | 0.68 | 33.10% | 3.35 | 8.14% | 1.30 |
| - Poland | 1630 | 2.66 | 8.78% | 12.22% | 0.32 | 0.85% | 0.32 | 1.08% | -7.60 |
| - Portugal | 1116 | 2.36 | 13.63% | 53.78% | 0.14 | 38.23% | 0.11 | 6.19% | 3.50 |
| - Slovenia | 1158 | 3.04 | 8.96% | 64.15% | 0.64 | 62.60% | 0.38 | 8.44% | 2.00 |
| - Spain | 1643 | 2.27 | 23.07% | 66.85% | 0.34 | 20.18% | 0.33 | 8.53% | 11.10 |
| - Sweden | 1338 | 2.63 | 7.68% | 69.48% | 2.93 | 38.69% | 9.80 | 11.63% | -0.10 |
| - Switzerland | 1058 | 2.94 | 4.70% | 58.35% | 3.27 | 24.56% | 4.03 | 11.18% | 0.90 |
| - United Kingdom | 1618 | 2.82 | 6.61% | 32.03% | 0.61 | 21.40% | 0.62 | 8.33% | -0.60 |

| Table A2. Standardized factor loadings and posterior probability intervals (PPI) for attitudes towards asylum policy on both levels (N=32320)^[[1]](#footnote-1)^ | | | | |
| --- | --- | --- | --- | --- |
|  | **Within** | | **Between** | |
|  | Factor loading | 95% PPI | Factor loading | 95% PPI |
| **Gvrfgap** - The government should be generous in judging people’s applications for refugee status. | 0.776 | [0.673 to 0.881] | 0.805 | [0.534 to 0.972] |
| **Rfgbfml** - Refugees whose applications are granted should be entitled to bring in their close family members. | 0.561 | [0.490 to 0.644] | 0.917 | [0.647 to 0.995] |

| Table A3. Standardized factor loadings and posterior probability intervals (PPI) universalism and conformity-tradition (N=32548)^[[2]](#footnote-2)^ | | | | |
| --- | --- | --- | --- | --- |
|  | **Conformity-tradition** | | **Universalism** | |
|  | Factor loading | 95% PPI | Factor loading | 95% PPI |
| - **Ipmodst** - It is important to him/her to be humble and modest. He/she tries not to draw attention to himself. | 0.465 | [0.453 to 0.479] |  |  |
| - **Ipfrule** - He/she believes that people should do what they're told. He/she thinks people should follow rules at all times, even when no-one is watching. | 0.525 | [0.511 to 0.538] |  |  |
| - **Ipbhprp** - It is important to him/her always to behave properly. He/she wants to avoid doing anything people would say is wrong. | 0.714 | [0.701 to 0.725] |  |  |
| - **Imptrad** - Tradition is important to him/her. He/she tries to follow the customs handed down by his/her religion or his/her family. | 0.461 | [0.445 to 0.472] |  |  |
| **Ipeqopt** - He/she thinks it is important that every person in the world should be treated equally. He/she believes everyone should have equal opportunities in life. |  |  | 0.586 | [0.575 to 0.601] |
| **Ipudrst** - It is important to him/her to listen to people who are different from him/her. Even when he/she disagrees with them, he/she still wants to understand them. |  |  | 0.576 | [0.564 to 0.587] |
| - **Impenv** - He/she strongly believes that people should care for nature. Looking after the environment is important to him/her. |  |  | 0.567 | [0.555 to 0.581] |
| - **Correlation** | 0.485 | | | |

X^2^=1310.374; df=11; CFI=0.945; TLI=0.896; RMSEA=0.060; SRMR=0.036

| Table A4. Robustness checks with different operationalisations of the contextual predictors^[[3]](#footnote-3)^ | | |
| --- | --- | --- |
|  | **Attitudes towards asylum policy** | |
|  | Estimate | 95% PPI |
| **MODEL 1** |  |  |
| Average number asylum applications 2015-2016 | -0.121 | [-0.584 to 0.369] |
| % conflict regions | -0.158 | [-0.675 to 0.406] |
| % unemployment | -0.636* | [-0.976 to -0.145] |
| Recognition rate | 0.422 | [-0.183 to 0.921] |
|  |  |  |
| **MODEL 2** |  |  |
| % foreign-born | 0.498 | [-0.078 to 0.927] |
| % conflict regions | -0.020 | [-0.524 to 0.506] |
| % unemployment | -0.500* | [-0.800 to -0.099] |
| Recognition rate | -0.079 | [-0.542 to 0.506] |
|  |  |  |
| **MODEL 3** |  |  |
| Number asylum applications | 0.052 | [-0.432 to 0.518] |
| % conflict regions | -0.212 | [-0.775 to 0.428] |
| Difference unemployment rate 2006-2016 | -0.458 | [-0.873 to 0.079] |
| Recognition rate | 0.494 | [-0.203 to 1.035] |

PPI: posterior probability interval; *P-value < 0.05

| Table A5. Standardized parameter estimates and posterior probability intervals of a model predicting attitudes towards immigration policy (N=31597)^[[4]](#footnote-4)^ | | |
| --- | --- | --- |
|  | **Attitudes towards immigration policy** | |
|  | Estim. | 95% PPI |
| **INDIVIDUAL VARIABLES** |  |  |
| **Gender** |  |  |
| Female (ref) |  |  |
| Male | 0.004 | [-0.005 to 0.014] |
| **Age** | 0.097* | [0.086 to 0.108] |
| **Education** |  |  |
| Lower (secondary) | 0.021* | [0.010 to 0.032] |
| Higher secondary (ref) |  |  |
| Tertiary | -0.020* | [-0.031 to -0.008] |
| **Subjective** **income** |  |  |
| Comfortable (ref) |  |  |
| Coping | 0.009 | [-0.002 to 0.020] |
| Difficult | 0.033* | [0.022 to 0.044] |
| Very difficult | 0.040* | [0.030 to 0.050] |
| **Occupation** |  |  |
| Service | -0.013* | [-0.024 to -0.002] |
| Blue collar (ref) |  |  |
| White collar | -0.030* | [-0.044 to -0.016] |
| Self-employed | -0.007 | [-0.018 to 0.004] |
| Unemployed | -0.007 | [-0.018 to 0.004] |
| Retired/non-active | -0.061* | [-0.076 to -0.046] |
| **Religiosity** | -0.018* | [-0.029 to -0.006] |
| **Area of residence** |  |  |
| Rural area (ref) |  |  |
| Big city, suburbs or town | -0.005 | [-0.014 to 0.005] |
| **Perceived safety** |  |  |
| Not safe (ref) |  |  |
| Safe | -0.014* | [-0.024 to -0.004] |
| **Conformity-tradition** | 0.188* | [0.168 to 0.208] |
| **Universalism** | -0.234* | [-0.252 to -0.215] |
| **Economic threat** | 0.298* | [0.285 to 0.311] |
| **Cultural threat** | 0.268* | [0.254 to 0.282] |
| **R² (within)** | 0.475 | |
| **CONTEXTUAL VARIABLES** |  |  |
| **Number asylum applications** | -0.248 | [-0.627 to 0.212] |
| **% conflict regions** | 0.340 | [-0.229 to 0.789] |
| **% unemployment** | -0.048 | [-0.439 to 0.360] |
| **Recognition rate** | -0.444 | [-0.857 to 0.127] |
| **R² (between)** | 0.273 | |

PPI: posterior probability interval; *P-value < 0.05; X^2^=2241.085; df=160; CFI=0.939; TLI=0.901; RMSEA=0.020; SRMR_W_=0.029; SRMR_B_=0.138

1. The fit of this model cannot be assessed, as it is just identified and thus automatically yields perfect fit with the data [↑](#footnote-ref-1)
2. The modification indices showed that allowing error correlations between certain items is necessary. Error correlations between the items that state that people should care for nature and that traditions should be maintained, and between items that state that people should not draw attention to themselves and that people should listen to others, are allowed. These correlations might be due to their interrelatedness in a religious context and because they are consecutive items in the questionnaire [↑](#footnote-ref-2)
3. Only the results from the contextual predictors are portrayed here, as the individual-level results do not change when including alternative country-level indicators [↑](#footnote-ref-3)
4. Only the direct effects on attitudes towards immigration policy are displayed here, as the effects of the structural variables on the human values and the threat perceptions do not change considerably. [↑](#footnote-ref-4)
